# CompARE: study protocol for a phase III randomised controlled platform trial comparing alternative regimens for escalating treatment of intermediate and high-risk oropharyngeal cancer

**DOI:** 10.1186/s13063-023-07881-1

**Published:** 2024-01-15

**Authors:** Hisham Mehanna, Piers Gaunt, Anthony Kong, Andrew Hartley, Paul Sanghera, Martin Forster, Mehmet Sen, Vinidh Paleri, Charles Fong, Dinos Geropantas, Devraj Srinivasan, Satya Garikipati, Rafael Moleron, Georgina Casswell, Eleanor Aynsley, Amy Ward, Lorcan O’Toole, Arafat Mirza, Charlotte Firth, Isla Humphreys, Tessa Fulton-Lieuw, Tom Roques, Paul Nankivell

**Affiliations:** 1 Institute of Head and Neck Studies and Education (InHANSE), Institute of Cancer and Genomic Sciences, University of Birmingham, B15 2TT, Birmingham, UK; 2 Cancer Research UK Clinical Trials Unit, Institute of Cancer & Genomic Sciences, University of Birmingham, Birmingham, UK; 3 King’s College London, London, UK; 4 Queen Elizabeth Hospital, Birmingham, UK; 5 UCL Cancer Institute, UCLH, London, UK; 6 St James’s University Hospital, Leeds, UK; 7 The Royal Marsden, London, UK; 8 Norfolk and Norwich University Hospitals NHS Foundation Trust, Norwich, UK; 9 Western General Hospital, Edinburgh, UK; 10 Weston Park Hospital, Sheffield, UK; 11 Aberdeen Royal Infirmary, Aberdeen, UK; 12 University Hospitals Bristol NHS Foundation Trust, Bristol, UK; 13 The James Cook University Hospital, Middlesbrough, UK; 14 Queen’s Hospital Romford, London, UK; 15 Castle Hill Hospital, Yorkshire, UK; 16 Royal Preston Hospital, Preston, UK

**Keywords:** Clinical trial, Adaptive multi-arm multi-stage design, Oropharyngeal cancer, QuinteT recruitment intervention

## Abstract

**Background:**

Patients with intermediate and high-risk oropharyngeal cancer (OPC) have poorer response to standard treatment and poorer overall survival compared to low-risk OPC. CompARE is designed to test alternative approaches to intensified treatment for these patients to improve survival.

**Methods:**

CompARE is a pragmatic phase III, open-label, multicenter randomised controlled trial with an adaptive multi-arm, multi-stage design and an integrated QuinteT Recruitment Intervention. Eligible OPC patients include those with human papillomavirus (HPV) negative, T1–T4, N1–N3 or T3–4, N0, or HPV positive N3, T4, or current smokers (or ≥ 10 pack years previous smoking history) with T1–T4, N2b–N3. CompARE was originally designed with four arms (one control [arm 1] and three experimental: arm 2—induction chemotherapy followed by arm 1; arm 3—dose-escalated radiotherapy plus concomitant cisplatin; and arm 4—resection of primary followed by arm 1). The three original experimental arms have been closed to recruitment and a further experimental arm opened (arm 5—induction durvalumab followed by arm 1 and then adjuvant durvalumab). Currently recruiting are arm 1 (control): standard treatment of 3-weekly cisplatin 100 mg/m^2^ or weekly 40 mg/m^2^ with intensity-modulated radiotherapy using 70 Gy in 35 fractions ± neck dissection determined by clinical and radiological assessment 3 months post-treatment, and arm 5 (intervention): one cycle of induction durvalumab 1500 mg followed by standard treatment then durvalumab 1500 mg every 4 weeks for a total of 6 months. The definitive and interim primary outcome measures are overall survival time and event-free survival (EFS) time, respectively. Secondary outcome measures include quality of life, toxicity, swallowing outcomes, feeding tube incidence, surgical complication rates, and cost-effectiveness. The design anticipates that after approximately 7 years, 84 required events will have occurred to enable analysis of the definitive primary outcome measure for this comparison. Planned interim futility analyses using EFS will also be performed.

**Discussion:**

CompARE is designed to be efficient and cost-effective in response to new data, emerging new treatments or difficulties, with the aim of bringing new treatment options for these patients.

**Trial registration:**

ISRCTN ISRCTN41478539. Registered on 29 April 2015

**Supplementary Information:**

The online version contains supplementary material available at 10.1186/s13063-023-07881-1.

## Administrative information

Note: The numbers in curly brackets in this protocol refer to the SPIRIT checklist item numbers. The order of the items has been modified to group similar items (see http://www.equator-network.org/reporting-guidelines/spirit-2013-statement-defining-standard-protocol-items-for-clinical-trials/).

**Table:** 

Title {1}	CompARE: study protocol for a phase III randomised controlled platform trial comparing alternative regimens for escalating treatment of intermediate and high-risk oropharyngeal cancer
Trial registration {2a and 2b}	EudraCT Number: 2014-003389-26 ISRCTN 41478539
Protocol version {3}	Version 8.0b, 02 June 2020
Funding {4}	This trial is supported by Cancer Research UK (C19677/A17226); CRUK trial number CRUK/13/026. Arm 5 is also supported by an academic unrestricted grant from AstraZeneca, who are also providing durvalumab free of charge. Cancer Research UK and AstraZeneca did not play any role in the design of the study and collection, analysis, and interpretation of the data. Cancer Research UK also did not play any role in writing this manuscript. AstraZeneca reviewed the manuscript prior to submission; no changes were made.
Author details {5a}	HM, TFL, PN: Institute of Head and Neck Studies and Education (InHANSE), Institute of Cancer and Genomic Sciences, University of Birmingham, Birmingham, UK PG, CF, IH: Cancer Research UK Clinical Trials Unit, Institute of Cancer & Genomic Sciences, University of Birmingham, Birmingham, UK AK: King’s College London, London, UK AH, PS, CF: Queen Elizabeth Hospital, Birmingham, UK MS: St James’s University Hospital, Leeds, UK MF: UCL Cancer Institute, UCLH, London, UK VP: The Royal Marsden, London, UK DG, TR: Norfolk and Norwich Hospital, Norfolk, UK DS: Western General Hospital, Edinburgh, UK SG: Weston Park Hospital, Sheffield, UK RM: Aberdeen Royal Infirmary, Aberdeen, UK GC: University Hospital Bristol, Bristol, UK EA: The James Cook University Hospital, Middlesbrough, UK AW: Queen’s Hospital Romford, London, UK LOT: Castle Hill Hospital, Yorkshire, UK AM: Royal Preston Hospital, Preston, UK
Name and contact information for the trial sponsor {5b}	Research Governance & Integrity, Research Strategy and Services Division, Research Park, Birmingham, B15 2TT, UK. Email: researchgovernance@contacts.bham.ac.uk
Role of sponsor {5c}	The trial sponsor has had no input to the study design; collection, management, analysis, and interpretation of the data; or writing of any trial reports or documents.

## Introduction

### Background and rationale {6a}

The incidence of oropharyngeal cancer (OPC) has increased dramatically over the last 30 years, with the rapid rise of OPC largely due to the role of human papillomavirus (HPV) infection in carcinogenesis. In recent meta-analyses, it has been shown that the proportion of OPC caused by HPV has more than doubled [1, 2].

Response of OPC to standard-of-care treatment can be divided into favourable and poor prognostic groups according to whether there is an association with HPV and with smoking. HPV status of OPC can be accurately defined using a combination of high-risk HPV polymerase chain reaction (PCR) or in situ hybridisation and p16 expression by immunohistochemistry [3]. HPV-negative OPC has a much worse outcome than HPV-positive OPC (2-year overall survival (OS) outcome 50–60%, versus 80–95% respectively). The seminal study in this field by Ang et al. showed that the prognostic value of HPV status can be further improved by combining it with smoking status, tumour size, and nodal stage [4]. Critically, this study identified three separate risk classifications: low-risk (3-year OS = 93%), intermediate-risk (3-year OS = 70.8%), and high-risk (3-year OS = 46%). The details of each risk group are given in Table 1.

**Table 1:** Oropharyngeal cancer risk categories

Risk group	Definition	3-year OS (95% CI)	3-year LRC rate	UK incidence: proportion of OPC cases	Projected no. per year
**Low-risk**	HPV+ve non-smokers (or smoke < 10 pack years) with small nodal disease (N0–N2a) and T1–3 tumours	93% (88.3–97.7)	90.4	33%	596
**Intermediate-risk**	HPV+ve with advanced nodal disease (N2b/c, N3) and > 10 pack year smoking or T4 tumour HPV-ve non-smokers with T1–3 tumours	70.8% (60.7–80.8)	80.9	47%	829
**High-risk**	HPV-ve smoking associated with T4 tumour	46% (34.7–57.7)	57.3	20%	343

*CI* Confidence interval, *HPV* Human papillomavirus, *LRC* Loco-regional control, *OPC* Oropharyngeal cancer, *OS* Overall survival

The results of the Ang et al. prognostic classification have now been replicated by others [5].

As a result of Ang et al.’s study, prognostic classification and new treatment paradigms for HPV-positive and HPV-negative OPC were proposed. For patients with low-risk disease, new treatment strategies aim to improve the toxicity profile by using less intensive chemotherapy or radiotherapy regimens. However, the De-ESCALaTE and Radiation Therapy Oncology Group (RTOG) 1016 trials demonstrated clear evidence of detriment to loco-regional control (LRC) when substituting cisplatin for cetuximab therapy in low-risk OPC patients [6, 7], thus arguing against de-escalation in this group. Conversely, the poor outcomes of patients with intermediate-risk HPV-positive and with high-risk HPV-negative disease (as per the Ang et al. classification) suggest that they may benefit from intensification of treatment to improve outcomes. Brotherston et al. have shown that OPC patients are unwilling to trade survival for reduced toxicity [8]. Ang’s analysis shows that the differences in survival between the low-, intermediate-, and high-risk groups when treated with chemoradiotherapy are mainly due to differences in LRC, which at 3 years were 90.4%, 80.9%, and 57.3%, respectively [4]. Huang et al. showed that the rate of distance metastasis was the same for HPV-positive and HPV-negative cases [9]. This suggests that a more aggressive treatment approach, especially one that aims to increase LRC, in the intermediate- and high-risk groups may improve outcomes significantly. Consequently, new treatment paradigms are being considered for both intermediate- and high-risk OPC, which is the focus of this trial.

### Objectives {7}

The primary trial objective for CompARE is to examine the outcomes of alternative treatments, aiming to improve overall survival time in intermediate- and high-risk OPC.

The secondary objectives are to compare the quality of life (QoL), toxicity outcomes, and swallowing function of these alternative treatments. Additional objectives relating to qualitative recruitment, health economics and translational research are listed in Table 2.

**Table 2:** Additional objectives for the CompARE trial

**Qualitative Recruitment Investigation (QRI) objectives**	1. To monitor recruitment rates and identify sources of recruitment difficulties in the first year of the trial 2. To develop a plan to optimise randomisation and informed consent
**Health economics objectives**	1. To compare cost-effectiveness in all treatment arms through cost-utility analysis 2. To estimate the cost per quality-adjusted life years (QALY) over the 2-year period of the trial
**Translational research (CompARE Collect) objectives**	1. To prospectively collect and ‘bank’ high-quality tissue, saliva, and blood samples from patients with intermediate- and high- risk OPC 2. To develop and validate biomarker classifiers to aid better stratification of treatment selection 3. To develop several inter-related and complementary head and neck translational research projects

### Trial design {8}

**Fig. 1:**
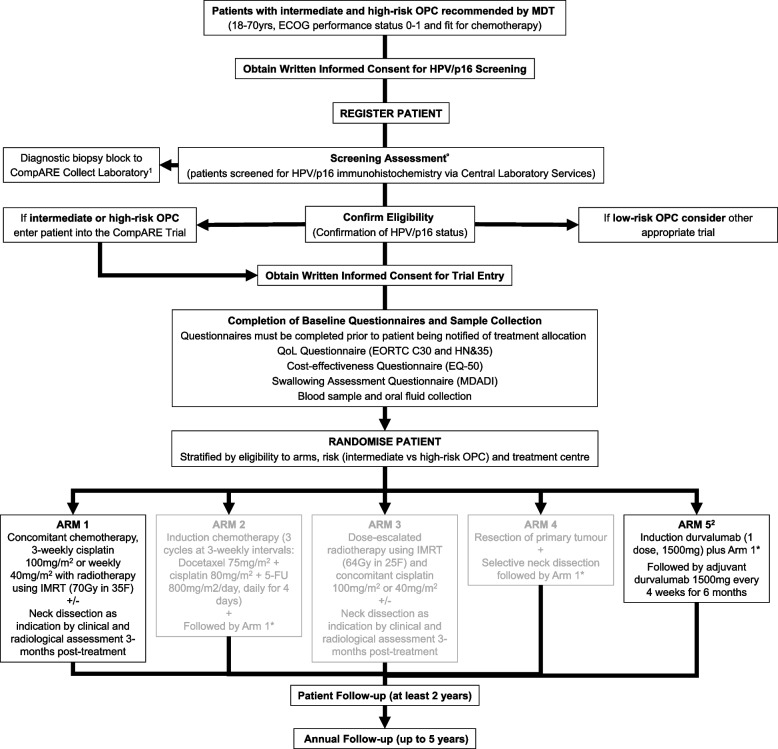
CompARE trial design. CompARE is a pragmatic phase III open-label multicenter randomised controlled trial with an adaptive multi-arm multi-stage design. Recruitment remains open for arms 1 and 5. Recruitment was suspended to arm 2 on 9 January 2017, recruitment suspended to arm 3 on 12 September 2019, and recruitment suspended to arm 4 on 7 February 2019. ^1^Samples collected for translational research (CompARE Collect) if the patient has consented. ^2^Additional baseline tests (clinical chemistry) are required for patients randomised to arm 5. *Neck dissection is required if a persistent disease is identified in the neck on clinical and radiological imaging at 3 months post-treatment. °Screening assessment: confirmation of HPV/p16 status should be provided by central laboratory services. For randomisation purposes, local p16 test results can be used; however, the diagnostic biopsy sample must still be sent to central laboratory services for confirmation. 5-FU, 5-fluorouracil; ECOG, Eastern Cooperative Oncology Group; HPV, human papillomavirus; IMRT, intensity-modulated radiotherapy; MDT, multidisciplinary team; OPC, oropharyngeal cancer

CompARE is a multicentre, phase III open-label randomised controlled platform trial using an efficient, adaptive, multi-arm multi-stage (MAMS) design. It incorporates a QuinteT Recruitment Intervention (QRI) [10] aiming to optimise recruitment and consenting. Standard treatment (chemotherapy plus radiotherapy: arm 1) will be compared as a control to experimental arms of various modes of treatment intensification (Fig. 1).

## Methods: participants, interventions, and outcomes

### Study setting {9}

CompARE is being conducted across 37 UK hospital sites. The University of Birmingham is the trial sponsor, with the study coordinated and run by the Cancer Research UK Clinical Trials Unit (CRCTU). A list of study sites is provided in Additional file 1: Appendix 1.

### Eligibility criteria {10}

The current inclusion and exclusion criteria for the arm 1 versus arm 5 comparison are listed in Table 3.

**Table 3:** Eligibility criteria for the CompARE trial

Inclusion criteria	Exclusion criteria
1. Oropharyngeal squamous cell carcinoma (OPSCC) in the base of the tongue and tonsil (includes bilateral tumours) with a multidisciplinary team recommendation for treatment with definitive concurrent chemoradiotherapy 2. All OPC T4 or N3 (HPV-pos and HPV-neg) or all HPV-neg OPC T1–T4, N1–N3, or T3–4, N0 or HPV-pos OPC T1-T4 with N2b-N3 nodes and who are smokers ≥ 10 pack years current or previous smoking history 3. Minimum life expectancy of 3 months 4. Eastern Cooperative Oncology Group (ECOG) performance status 0–1 5. Body weight of > 30 kg 6. Adequate renal function, estimated glomerular filtration rate (eGFR) > 50 mL/min calculated using Cockcroft-Gault formula 7. Adequate bone marrow function (absolute neutrophil count (ANC) ≥ 1.5 × 109/L, haemoglobin ≥ 9.0 g/dL and platelets ≥ 100 × 109/L) 8. Adequate liver function, i.e. serum bilirubin ≤ 1.5 times the upper limit of normal (ULN), AST (SGOT)/ALT (SGPT) ≤ 2.5 × institutional upper limit of normal 9. Prothrombin time (PT) ≤ 1.5 × ULN or international normalised ratio (INR) ≤ 1.5 10. Magnesium ≥ lower limit of normal 11. No cancers in previous 5 years, except for basal cell carcinoma of the skin and cervical intra-epithelial neoplasia (CIN) 12. Aged 18–70 13. Written informed consent given for the trial 14. Surgically resectable disease if being randomised to all four arms 15. Evidence of post-menopausal status or negative urinary or serum pregnancy test for female pre-menopausal patients. Women will be considered post-menopausal if they have been amenorrheic for 12 months without an alternative medical cause. The following age-specific requirements apply: a. Women < 50 years of age would be considered post-menopausal if they have been amenorrheic for 12 months or more following cessation of exogenous hormonal treatments and if they have luteinising hormone and follicle-stimulating hormone levels in the post-menopausal range for the institution or underwent surgical sterilisation (bilateral oophorectomy or hysterectomy). b. Women ≥ 50 years of age would be considered post-menopausal if they have been amenorrheic for 12 months or more following cessation of all exogenous hormonal treatments, had radiation-induced menopause with last menses > 12 months ago, had chemotherapy-induced menopause with last menses > 12 months ago, or underwent surgical sterilisation (bilateral oophorectomy, bilateral salpingectomy, or hysterectomy). 16. Willingness to comply with the protocol for the duration of the study, including undergoing treatment and scheduled visits and examinations including follow-up	1. All T1–T2, N0 OPC (HPV-pos or HPV-neg) 2. HPV-positive patients who are T1–T3, N0–N2c non-smokers or T1–T3, N0–N2c smokers with ≤ 10 pack years or T1–T2, N0–N2a smokers with ≥ 10 pack years 3. Unfit for chemoradiotherapy regimens 4. Creatinine clearance < 50 mL/min 5. Treatment with any of the following, prior to randomisation: (a) any investigational medicinal products (IMP) within 30 days; (b) any other chemotherapy, immunotherapy, or anticancer agents within 3 weeks; (c) major surgical procedure (as defined by the investigator) within 4 weeks, unless for diagnostic purposes; and (d) concurrent use of hormonal therapy for non-cancer-related conditions (e.g. hormone replacement therapy is acceptable) 6. History of allergic reactions or hypersensitivity to any of the IMPs and excipients used in this trial 7. Uncontrolled intercurrent illness including, but not limited to, ongoing or active infection, symptomatic congestive heart failure, uncontrolled hypertension, unstable angina pectoris, cardiac arrhythmia, interstitial lung disease, serious chronic gastrointestinal conditions associated with diarrhoea, or any subject known to have evidence of acute or chronic hepatitis B, hepatitis C, human immunodeficiency virus (HIV), or psychiatric illness/social situations that would limit compliance with study requirement, substantially increase the risk of incurring AEs or compromise the ability of the subject to give written informed consent 8. Women who are pregnant or breast-feeding. Women of childbearing potential must have a negative pregnancy test performed within 7 days prior to randomisation 9. Men or women who are not prepared to practise methods of contraception of proven efficacy during treatment and for 6 months following the end of treatment 10. Any condition that, in the opinion of the investigator, would interfere with the evaluation of study treatment or interpretation of patient safety or study results 11. Additional exclusion criteria for arm 5 only 12. Any previous treatment with PD-L or PD-L1 inhibitor, including durvalumab 13. Current or prior use of immunosuppressive medication within 14 days before the first dose of durvalumab; the following are exceptions to this criterion: injections (e.g. intra articular injection), systemic corticosteroids at physiologic doses not to exceed 10 mg/day of prednisone or its equivalent, and steroids as premedication for hypersensitivity reactions (e.g. CT scan, premedication) 14. Active or prior documented autoimmune or inflammatory disorders including inflammatory bowel disease, e.g. colitis or Crohn’s disease, diverticulitis (with the exception of diverticulosis), systemic lupus erythematosus, Sarcoidosis syndrome, or Wegener syndrome (granulomatosis with polyangiitis, Graves’ disease, rheumatoid arthritis, hypophysitis, uveitis, etc.). The following are exceptions to this criterion: patients with vitiligo or alopecia, patients with hypothyroidism (e.g. following Hashimoto syndrome) stable on hormone replacement, any chronic skin condition that does not require systemic therapy, patients without active disease in the last 5 years may be included but only after consultation with the study physician, and patients with celiac disease controlled by diet alone 15. History of active primary immunodeficiency 16. Active infection including tuberculosis (clinical evaluation that includes clinical history, physical examination and radiographic findings, and TB testing in line with local practice), hepatitis B (known positive HBV surface antigen (HBsAg) result), hepatitis C, or human immunodeficiency virus (positive HIV 1/2 antibodies). Patients with a past or resolved HBV infection (defined as the presence of hepatitis B core antibody [anti-HBc] and absence of HBsAg) are eligible. Patients positive for hepatitis C (HCV) antibody are eligible only if polymerase chain reaction is negative for HCV RNA 17. History of allogeneic organ transplant 18. Receipt of live attenuated vaccination within 30 days prior to study entry or within 30 days of receiving durvalumab. Inactivated viruses, such as those in the influenza vaccine, are permitted.

### Who will take informed consent? {26a}

Potential patients are identified at the head and neck multidisciplinary team meeting in participating hospitals. Patients are approached in a ‘pre-screen’ fashion about their willingness to participate in the trial by the principal investigator and/or research nurse in the clinic and are given trial information. Patients are given a minimum of 24 h to read the information and ask questions before giving written consent. Exemplar patient information sheets along with summary sheet, and informed consent forms are included in Additional file 1: Appendices 2 and 3, respectively.

### Additional consent provisions for collection and use of participant data and biological specimens {26b}

CompARE Collect is an optional sub-study within the CompARE trial. This sub-study involves collection of formalin-fixed paraffin-embedded tissue for genetic analyses at diagnosis, at neck dissection, and recurrence or progression, and blood and oral fluid samples at baseline, end of chemoradiotherapy, and 3 months and 12 months after end of chemoradiotherapy treatment. A separate consent form is available for patients choosing to allow collection of their biological material (Additional file 1: Appendix 3).

### Interventions

#### Explanation for the choice of comparators {6b}

##### Arm 1: Concomitant cisplatin chemotherapy plus radiotherapy

The control arm consisted of concomitant chemoradiotherapy, 3-weekly cisplatin 100 mg/m^2^, or weekly 40 mg/m^2^ with intensity-modulated radiotherapy (IMRT) using 70 Gy in 35 fractions (F) ± neck dissection as indicated by clinical and radiological assessment 3 months post-treatment. The 3-weekly cisplatin regimen is the international gold standard. More recently, evidence supporting weekly cisplatin has been published [11].

#### Intervention description {11a}

##### Arm 2: Induction chemotherapy followed by arm 1

Induction chemotherapy (three cycles at 3-weekly intervals: docetaxel 75 mg/m^2^ + cisplatin 80 mg/m^2^ + 5-fluorouracil 800 mg/m^2^/day, daily for 4 days), followed by arm 1.

Recruitment was suspended to arm 2 on 9 January 2017 due to a combination of patients declining to participate due to the overall length of treatment as well as emerging evidence from other trials suggesting a lack of efficacy of other induction regimens [12, 13].

##### Arm 3: Dose-escalated radiotherapy plus concomitant cisplatin

Dose-escalated chemoradiotherapy using IMRT 64 Gy in 25 F + cisplatin 100 mg/m^2^ day 1 of week 1 and of week 5 or weekly 40 mg/m^2^ ± neck dissection as indicated by clinical and radiological assessment at 3 months post-chemoradiotherapy treatment.

Recruitment was suspended to arm 3 on 12 September 2019 (see the ‘Discussion’ section).

##### Arm 4: Resection of primary followed by arm 1

Resection of primary and selective neck dissection (within 4 weeks of randomisation to study) followed by chemoradiotherapy as per arm 1. For T1 and T2 primary tumours, resection had to be transoral. For T3 and T4 primary tumours, resection was recommended to be transoral if possible, otherwise by open surgery.

Recruitment was suspended to arm 4 on 7 February 2019 due to a lack of recruitment.

##### Arm 5: Induction durvalumab followed by arm 1 and then adjuvant durvalumab

One dose of induction durvalumab 1500 mg by intravenous (IV) infusion followed by arm 1 within 4 weeks. Within 1–2 weeks after the completion of arm 1 (up to a maximum of 6 weeks), adjuvant durvalumab 1500 mg is given every 4 weeks, for up to 6 months.

#### Criteria for discontinuing or modifying allocated interventions {11b}

Patients should discontinue trial treatment in the following circumstances:

- The patient chooses to discontinue treatment and/or terminate participation in the trial
- The investigator considers that continuation is not in the best interest of the patient
- Delay to treatment of more than 21 days in starting the next cycle of treatment due to toxicity
- Progressive disease according to clinical investigations or radiographic investigations
- Participant becomes pregnant, despite appropriate contraceptive measures
- Intent to become pregnant
- Suspension or termination of the trial by the sponsor
- One or more of the exclusion criteria has been met and continuing treatment may constitute a safety risk
- Dose-limiting toxicity
- Infusion reaction grade ≥ 3 following durvalumab administration
- Patient non-compliance that, in the opinion of the investigator or sponsor, warrants withdrawal, e.g. refusal to adhere to scheduled visits
- Initiation of alternative anti-cancer therapy including another investigational agent

Dose modification and toxicity management guidelines for immune-related, infusion-related, and non-immune-mediate reactions for durvalumab (arm 5) are detailed in Additional file 1: Appendix 4.

### Strategies to improve adherence to interventions {11c}

Compliance to radiotherapy (IMRT) and chemotherapy is being reported. Furthermore, radiotherapy must be delivered via IMRT only, conforming to the CompARE radiotherapy quality assurance volumetric outlining protocols. Toxicity management protocols are recommended to reduce unwanted side effects. These include hydration, antiemetics, pain management, and the management of other complications, e.g. myelosuppression and nephrotoxicity.

### Relevant concomitant care permitted or prohibited during the trial {11d}

Details of prohibited medications for arm 1 are listed in Additional file 1: Appendix 5. Details of prohibited medications for arm 5 are listed in Additional file 1: Appendix 6.

#### Provisions for post-trial care {30}

No specific provisions are made for post-trial care. Follow-up is directed according to local institutional guidelines.

### Outcomes {12}

The primary outcome measure for the definitive endpoint is OS time, and for the interim stages, EFS time.

The following are the secondary outcome measures:

1. Toxicity events—Total number of acute (< 3 months post-treatment) and late (> 3 months up to 2 years) severe (grades 3–5) toxicity events at 2 years post-randomisation will be measured using the Common Terminology Criteria for Adverse Events (CTCAE) version 4.0 and version 3.0 for scoring mucositis. RTOG Radiation Morbidity Scoring Criteria will be used to grade late side effects due to radiotherapy.
2. Overall and head and neck-specific QoL will be assessed at 24 months post-randomisation using the European Organisation for Research and Treatment of Cancer (EORTC) Quality of Life Questionnaire QLQ-C30 [14] and H&N35 [15] Questionnaires.
3. Swallowing outcomes—These will be assessed using the M.D. Anderson Dysphagia Inventory (MDADI) Questionnaire [16], at 24 months and percutaneous endoscopic gastrostomy (PEG) utilisation rates at 1 year.
4. Cost-effectiveness will be assessed using EuroQol Group (EQ-5D) [17] and primary and secondary resource utilisation data.
5. Surgical complication—Documented data derived from patient hospital and clinic case note files. Data will be reported separately for primary resections and neck dissections.

### Participant timeline {13}

**Fig. 2:**
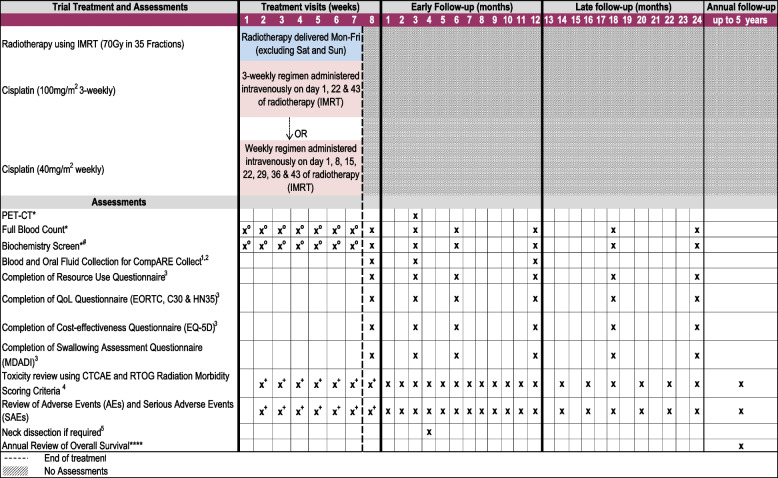
Schedule of events for arm 1 of the CompARE trial. Schedule of events for treatment arm 1, concomitant cisplatin chemotherapy plus radiotherapy. *Assessment which is part of standard practice. ****Patient followed up annually for survival data for up to 5 years. ^#^Biochemistry screen: alkaline phosphatase, alanine transferase, biocarbonate, calcium, creatinine, glomerular filtration rate, liver function tests, glucose, magnesium, potassium, sodium, total bilirubin, total protein, urea, or blood urea nitrogen. Serum or plasma analysis will include albumin, glucose, and gamma-glutamyl transferase. ^o^Cisplatin 100 mg/m^2^ 3-weekly: full blood count and biochemistry screen to be performed 3-weekly. Cisplatin 40 mg/m^2^ weekly: full blood count and biochemistry screen to be performed weekly. ^+^Toxicity and adverse events assessed during chemoradiotherapy. ^1^Samples collected if the patient has consented for CompARE Collect. ^2^Blood and oral fluid samples should also be collected at recurrence or progression (formalin-fixed paraffin-embedded tissue block or needle aspirate sample should also be collected if recurrence is confirmed by histology/cytology). ^3^Questionnaires to be completed by the patient in the clinic at defined visits. ^4^Toxicity will be reviewed using CTCAE version 4.0 and version 3.0 for scoring mucositis. The RTOG Radiation Morbidity Scoring Criteria will be used to grade late side effects due to radiotherapy. ^5^Neck dissection is required if persistent disease is identified in the neck on imaging (PET-CT or contrast CT or contrast MRI) at 3 months post-chemoradiotherapy treatment. The same modality PET CT or CT or MRI should be used for all arms

**Fig. 3:**
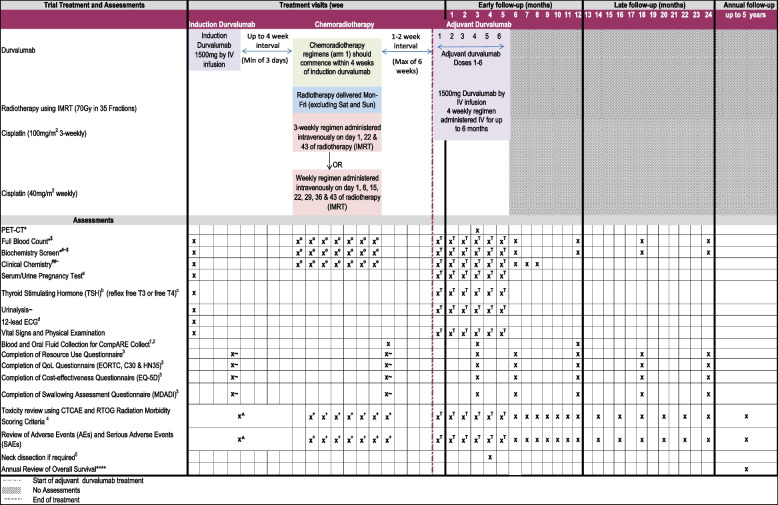
Schedule of events for arm 5 of the CompARE trial. Schedule of events for treatment arm 5, induction durvalumab plus arm 1 followed by adjuvant durvalumab. *Assessment part of standard practice. ****Patient followed up annually for survival data for up to 5 years. ^#^Biochemistry screen: alkaline phosphatase (ALP), alanine transferase (ALT), biocarbonate, calcium, creatinine, glomerular filtration rate, liver function tests (LFTs), glucose, magnesium, potassium, sodium, total bilirubin, total protein, urea, or blood urea nitrogen. Serum or plasma analysis will include albumin, glucose, and gamma-glutamyl transferase. Biochemistry screen must take place within 120 h prior to durvalumab infusion. ^##^Clinical chemistry screen: amylase, lactose dehydrogenase, and aspartate aminotransferase (AST). It is preferable that both amylase and lipase parameters are assessed. For sites where only one of these parameters is routinely measured then either lipase or amylase is acceptable. Clinical chemistry screen must take place within 120 h prior to durvalumab infusion. ^$^Results for LFTs, electrolytes, full blood count, and creatinine must be available before commencing an infusion (within 120 h) and reviewed by the treating physician or investigator prior to dosing. ^~^Tests for ALT, AST, ALP, and total bilirubin must be conducted and assessed concurrently. If total bilirubin is ≥ 2× upper limit of normal (and no evidence of Gilbert’s syndrome) then fractionate into direct and indirect bilirubin. ^a^For women of childbearing potential only. A urine or serum pregnancy test is acceptable. Women of childbearing potential are required to have a pregnancy test within 7 days prior to the first dose of study drug. ^b^If thyroid-stimulating hormone (TSH) is measured within 14 days prior to day 1 (first durvalumab infusion day), it does not need to be repeated at day 1. ^c^Free T3 or free T4 will only be measured if TSH is abnormal or if there is clinical suspicion of an AE related to the endocrine system. ^d^Any clinically significant abnormalities detected require triplicate ECG results. ^o^Cisplatin 100 mg/m^2^ 3-weekly: full blood count, biochemistry screen, and clinical chemistry screen to be performed 3-weekly. Cisplatin 40 mg/m^2^ weekly: full blood count, biochemistry screen, and clinical chemistry screen to be performed weekly. ^+^Toxicity and adverse events assessed during chemoradiotherapy. ^T^Assessments to be performed 4-weekly during adjuvant durvalumab treatment. ^Toxicity and adverse events assessed 2-weekly. ^1^Samples collected if the patient has consented for CompARE Collect. ^2^Blood and oral fluid samples should be collected at recurrence or progression (formalin-fixed paraffin-embedded tissue block or needle aspirate sample should also be collected if recurrence is confirmed by histology/cytology). ^3^Questionnaires to be completed by the patient in the clinic at defined visits. ^4^Toxicity will be reviewed using CTCAE version 4.0 and version 3.0 for scoring mucositis. The RTOG Radiation Morbidity Scoring Criteria will be used to grade late side effects due to radiotherapy. ^5^Neck dissection is required if persistent disease is identified in the neck on imaging (PET-CT or contrast CT or contrast MRI) at 3 months post-chemoradiotherapy treatment. The same modality PET CT or CT or MRI should be used for all arms. ^~^Questionnaires to be completed at the end of chemoradiotherapy and end of durvalumab treatment

A schedule of events for patients in arms 1 and 5 (those currently open) are included in Figs. 2 and 3, respectively.

### Sample size {14}

The study sample size is based on detecting a hazard ratio (HR) of 0.69 for OS. Based on reported data during the trial’s conception, a control survival proportion of 61% at 3 years is assumed. This was calculated by weighting the 3-year OS values of 71% and 46% for intermediate- and high-risk OPC patients, respectively, at a 60:40 ratio. Assuming exponential survival, a HR of 0.69 corresponds to an increase in 3-year OS rates from 61 to 71%.

The same HR will be applied to the interim assessment stages when analysing EFS. It is acknowledged that this is a conservative estimate to apply for EFS, as a larger treatment effect may be expected on EFS compared to OS. The proposed control for EFS at 1 year of 59% has been calculated in a similar fashion by weighting the same proportion of intermediate (3-year EFS of 65%) and high-risk (3-year EFS of − 50%) patients 60:40. A correlation of 0.6 has been assumed for the treatment effects for OS and EFS.

The initial trial design was a four-arm (three experimental versus one control with allocation ratio 2:1:1:1) trial performed using the N stage command in Stata. The trial was open for 2.25 years prior to arm 5 being added. The sample size determinations for the original comparisons and the arm 5 comparison were performed separately. The sample size determination for arm 5 versus arm 1 initially employed a 2:1 allocation ratio, which was revised to 1:1 as other trial arms closed. The power for the interim stages was 95% to minimise the chance of dropping an effective experimental arm. For the definitive outcome of OS, the power was 85%. The application of high power meant that less stringent alpha levels were to be applied. The one-sided significance levels applied in the design are 0.50 and 0.30 at each of our interim stages and 0.1 at the final stage. In designing a MAMS trial, assumptions must be made about the number of arms remaining in the study after each interim stage. However, for this comparison, as the sample size was separate, the assumption was that arm 5 would continue to recruit to the end of the study. The sample size assumed that 130 patients would be recruited per year and would take 6 years for the arm 5 versus arm 1 comparison. In practical terms, the recruitment projections will be reevaluated during the study using the artpep command in Stata to predict the actual recruitment timeframe and will be regularly assessed and discussed with the data monitoring committee (DMC). The sample size was reevaluated due to an increase in the proportion of intermediate-risk versus high-risk OPC patients (80:20). For this evaluation, the artpep command was used to account for patients already recruited and projected forward using 11 patients per month. The actual length of the trial and total number of patients recruited will depend on the observed recruitment rates, the observed event rates, and the number of treatment arms that pass successfully through the interim assessment stages. As recruitment to arm 5 commenced after the trial had been opened any arm 1 patients that were recruited prior to the commencement of arm 5 opening will not be incorporated into the analysis for arm 5 versus arm 1. Only contemporaneously recruited control patients in arm 1 will contribute to the arm 5 versus arm 1 analysis. Incorporating all of the sample size determinations across all the arms, the total duration for the CompARE study is predicted to be approximately 10 years with approximately 785 patients recruited in total.

### Recruitment {15}

The study opened to recruitment on 6 July 2015 and is expected to continue until at least January 2024.

Potential patients are identified at the head and neck multidisciplinary team meeting in participating hospitals. The trial incorporates a QRI to maximise trial recruitment and consenting during the first year of recruitment [10]. The aim of the QRI is to characterise and understand the success/failure of the trial recruitment process and provide timely guidance to the trial investigators to optimise recruitment. In addition, regular contact with sites, reviewing screening logs, and providing simplified summaries of the study have been incorporated to encourage recruitment. A steady-state recruitment of 10–11 patients per month during CompARE is being aimed for; each centre’s target was set in consultation with them according to their size, throughput, and previous trial activity. During the COVID-19 pandemic, recruitment was paused for 2 months between 18 March 2020 and 18 May 2020 then restarted.

### Assignment of interventions: allocation

#### Sequence generation {16a}

Eligible patients are randomised in a 1:1 ratio between the control arm (arm 1: standard treatment) and the durvalumab arms (arm 5). The allocation ratio for the control to experimental non-durvalumab arms (arms 2, 3, and 4) was 2:1. Randomisation is stratified by patient subgroup (patients with intermediate versus high-risk OPC) and treatment centre.

#### Concealment mechanism {16b}

Following completion of an online trial eligibility checklist, randomisation is performed via a computerised minimisation algorithm by staff at the CRCTU, University of Birmingham.

#### Implementation {16c}

When the trial was recruiting to the surgery arm, patients deemed suitable for surgery were offered all open treatment arms. Patients who did not wish to have surgery or were deemed ineligible for surgery or where the centre cannot offer surgery were offered the open non-surgical arms only. This was incorporated into the randomisation stratification. Furthermore, when other interventional arms were open, patients who do not meet the additional eligibility criteria for arm 5 or are recruited in centres where arm 5 was not yet activated, were randomised between the other open arms dependent on their status above.

### Assignment of interventions: blinding

#### Who will be blinded {17a}

Not applicable. This is an open-label trial.

#### Procedure for unblinding if needed {17b}

Not applicable. This is not a blinded trial.

### Data collection and management

#### Plans for assessment and collection of outcomes {18a}

Registration of patients enrolling on the trial will be performed by each hospital site using the online electronic Remote Data Capture (eRDC) system, after obtaining informed consent. At the end of the registration process, the patient will be allocated a unique patient trial number (TNO). The TNO will be used to identify the patient and will be recorded on the case report forms (CRFs), questionnaires and on any trial correspondence. Once assigned a TNO, all patient information recorded on the eRDC will be anonymised. Throughout the trial, all clinical data will be collected by the staff who are trained and competent to perform the role as detailed in the delegation log and approved by each site principal investigator.

### Plans to promote participant retention and complete follow-up {18b}

No specific plans are being implemented as the patient follow-up pathway is identical to that employed in standard clinical practice where patients are seen regularly on follow-up and adhere to this schedule. Data returns are promoted by regular communications between the trial team and the recruiting sites.

### Data management {19}

Trial research staff check incoming data submitted via remote data capture for compliance with the protocol, data consistency, missing data, and timing. Sites are asked to clarify missing data, inconsistencies, or discrepancies. Sites may be suspended from further recruitment in the event of serious and persistent non-compliance with the protocol and/or Good Clinical Practice (GCP), and/or poor recruitment. Any major problems identified during monitoring, including serious breaches of GCP and/or the trial protocol, are reported to the Trial Management Group (TMG), Trial Steering Committee (TSC), and the relevant regulatory bodies. Sites are also requested to notify the applicable National Coordinating Centre of any inspections by the relevant Competent Authority and to notify the UK Coordinating Centre of any significant audit findings.

All trial records must be archived and securely retained for at least 25 years. No documents will be destroyed without prior approval from the UK Coordinating Centre Document Storage Manager.

The CRCTU will hold the final trial dataset, and a data access committee will be responsible for the controlled sharing of anonymised clinical trial data with the wider research community to maximise potential patient benefit whilst protecting the privacy and confidentiality of trial participants. Data anonymised in compliance with the Information Commissioner’s Office requirements will be available for sharing with researchers outside of the trials team within 12 months of the primary publication.

### Confidentiality {27}

Data are handled and stored in accordance with the relevant data protection legislation in the applicable country. Patients are identified using only their unique trial number in correspondence between the applicable National Coordinating Centre and participating sites. However, if local regulation permits, patients are asked to consent to a non-anonymised copy of their signed informed consent form being sent to the National Coordinating Centre for in-house monitoring of consent.

Local investigators must maintain documents not for submission to the National Coordinating Centre in strict confidence. The National Coordinating Centres maintain the confidentiality of all patient data and will not disclose identifiable information to any third party other than those directly involved in the treatment of the patient and organisations for which the patient has given explicit consent for data transfer.

### Plans for collection, laboratory evaluation, and storage of biological specimens for genetic or molecular analysis in this trial/future use {33}

The collection of blood, oral fluid and tissue samples is optional for patients participating in the optional CompARE Collect sub-study. Formalin-fixed paraffin-embedded tissue is collected (subject to consent) for genetic analyses, including immunohistochemistry, in situ hybridisation, PCR, and other assays. Samples will be from diagnosis, neck dissection specimens, and recurrence or progression (if occurs). All samples will be collected in accordance with national regulations and requirements including standard operating procedures for logistics and infrastructure. Samples will be taken in appropriately licenced premises, stored, and transported in accordance with the Human Tissue Authority guidelines and NHS trust policies.

## Statistical methods

### Statistical methods for primary and secondary outcomes {20a}

All analyses will be via intention-to-treat (ITT) analyses, with all patients analysed in the arm to which allocated at randomisation.

### Primary outcome measures

For the purposes of this trial, OS is defined as the interval in whole days between the date of randomisation and the date of death from any cause. Follow-up for survival and recurrence will be repeated annually by the site as well as maintained through Office of National Statistics (ONS) tagging if required, for the duration of the trial. Patients who have not died at the time of analysis will be censored at the date when they were last known to be alive.

The median OS time and 3-year OS rate for each arm will be reported in addition to the HR (and confidence intervals) for the comparison of each arm to the control arm. The 3-year OS rate and the median OS will be calculated using the Kaplan-Meier method of estimation and presented with confidence intervals. OS will be compared for each treatment arm to the control arm using a stratified log-rank test. In addition to this analysis, a secondary analysis of OS will be undertaken using a Cox proportional hazards model adjusting for other potential prognostic factors including performance status, tumour size (T stage), and nodal stage combined p16 and HPV status. If non-proportional hazards are observed, then models accounting for non-proportional hazards will be considered and explored. As a sensitivity analysis, we will also report the results of an unadjusted log-rank test. Due to delayed treatment effects for the immunotherapy arm (arm 5), it might be necessary for the assessment of this arm compared to the control arm to be treated differently compared to the other experimental versus control comparisons, as the proportional hazards assumption might not be valid.

The interim outcome measure is EFS as defined as the interval in whole days between the date of randomisation and the date of a contributing event. Patients who are alive and event-free at the time of analysis will be censored at the date when they were last known to be alive and event-free. EFS will be compared for each treatment arm to the control arm using a stratified log-rank test. As for OS, a modelling approach to the analysis for EFS will be undertaken with a Cox proportional hazards model adjusting for prognostic factors. If non-proportional hazards are observed, then other models will be considered. As for the OS outcome, follow-up for events will be repeated annually by the site as well as maintained through ONS tagging for the duration of the trial. Any second primary tumour outside the head/neck area will not be considered an event. The bullet points below define scenarios that will be considered as events for EFS:

1. Death
2. Distant metastasis: Any distant metastasis will be considered as an event at the time it is detected. Clear evidence of distant metastases (lung, bone, brain, etc.) must be demonstrated to document distant metastasis. A biopsy is recommended where possible. A solitary, spiculated lung mass/nodule is considered a second primary neoplasm unless proven otherwise:
  - For the primary site:
    Any persistent disease 3 months after completion of treatment (at the 3-month evaluation time point) that necessitates salvage surgery will be considered as an event at the date of the confirmatory biopsy prior to the salvage surgery (if the biopsy date is not available then the salvage surgery date will apply).
    Any persistent/residual disease suspicious at the 3 months after completion of treatment (at the 3-month evaluation time point) that is confirmed by a subsequent serial scan will be considered as an event at the 3-month scan date.
    Any persistent disease 3 months after completion of treatment (at the 3-month evaluation time point) that dictates that the patient will be referred to palliative treatment (systemic chemotherapy or immunotherapy) or symptomatic management will be considered as an event at the 3-month date scan.
    Any recurrence, relapse, or progression (clinical or radiological) at any time. These events will be considered as an event at the date of recurrence or progression.
    Any new second primary will be considered as an event at the time it is detected.
  - For neck disease:
    Any persistent nodal disease detected at the 3-month time point treated with a neck dissection will be considered an event at the date of neck dissection if any of the excised nodes are histologically positive or uncertain. If all excised nodes are histologically negative, then this will not be considered as an event.
    Any persistent nodal disease detected at the 3-month time point treated as inconclusive and confirmed with subsequent serial scan followed by investigations and/or neck dissection will be considered an event at the 3-month scan post-chemoradiotherapy.
    Any recurrence, relapse, or progression in nodal disease will be considered as an event at the time it is detected.

In the interim and definitive analyses for each of the experimental arms to the control arm only contemporaneous patients that were entered into Strata® that contained both arms will be included in the analysis (termed eligible comparison).

### Secondary outcome measures

#### Toxicity

The total number of acute (< 3 months post-treatment) and late (> 3 months, up to 2 years) severe (grade 3 to 5) CTCAE events will be summarised using appropriate statistics. The numbers will be compared for each arm to the control arm using appropriate methodology. In addition, the number of patients experiencing one or more severe CTCAE events will be compared between each arm and the control arm.

#### Quality of life

The symptom and function scores will be calculated for each of the EORTC QLQ-C30 and H&N35 questionnaires returned. The overall global score from the EORTC questionnaires and the EQ-5D score will be compared between arms and analysed using longitudinal methods with consideration being given to missing data. The symptom and function scores will be presented graphically and compared across the important assessment time points. The balance between QoL and survival may be analysed using a quality-adjusted survival analysis.

#### Swallowing outcomes and PEG utilisation rates

Swallowing outcomes will be assessed using the MDADI questionnaire. The questionnaire returns emotional, physical, and functional scores as well as a global score. These scores will be determined using a scoring manual and assessed using longitudinal methods. The minimum clinically relevant difference is 10 points. PEG utilisation rates will be reported as a proportion of the respective time points when the information is collected.

#### Cost-effectiveness

Using differences in effectiveness (in terms of quality-adjusted survival) and costs between each treatment arm and the control arm, an appropriate cost-effectiveness analysis will be conducted.

#### Surgical complication rates

A count of surgical complication rates will be compiled for each patient that has received surgery. Relevant summary statistics will be presented for this data per treatment arm. It is anticipated that this data will be non-normal so the non-parametric Mann-Whitney test will be used to compare each experimental arm to the control arm. However, if the data are normal then the *t*-test will be employed to compare the relevant arms. Note that data will be reported separately for primary resection and neck dissections.

### Interim analyses {21b}

Planned interim analyses will be performed for the independent DMC and to conduct the interim futility assessments to determine whether an experimental arm should continue in the trial. The plan is to present data to the DMC after 6 months of recruitment and then annually during the recruitment phase; however, the timing of each analysis will be driven by the number of control events. The trial has been designed so that these control events should occur approximately annually and therefore coincide with the timing for a DMC meeting. The timings of meetings will be pragmatic.

Due to delayed treatment effects for the durvalumab arm (arm 5), it might be necessary for the assessment of this arm compared to the control arm to be treated differently compared to the other experimental versus control comparisons, as the proportional hazards assumption might not be valid [18]. Additional precautionary steps to be undertaken in the evaluation of arm 5 compared to the control arm are detailed in the statistical analysis plan.

Where the term ‘eligible comparison’ is used, this means that only the patients that undergo randomisation between the control arm and the experimental arm being evaluated will be used in the comparison. As arm 5 was introduced after the trial opened, then the timing of the analyses to compare that to the control arm may not align will the timings for the other arms. These analyses will only be conducted once the required numbers of control events have been observed for each comparison. Therefore, there may be a requirement to arrange a DMC meeting for the sole purpose of discussing the data for arm 5. The interim assessments for arm 5 will be carried out when 45 (first stage) and 75 (second stage) contemporaneous control (arm 1) EFS events have been observed.

Given the suspension to arm 3, the number of events required to trigger analyses will not change; however, should arm 3 be reopened, then it will be longer before this number of events is observed to make those determinations. The predicted timings of analyses will be monitored and presented to the DMC at regular meetings. For the arm 3 comparison, provided it is reopened, once 72 control EFS events (for the ‘eligible comparison’) have been recorded, then this will trigger the analysis for stage I; 116 control EFS events are required for stage II. The MAMS sample size calculation predicts the approximate timeframe when these interim stages will occur. With the change in allocation ratio for arm 5 (leading to fewer patients allocated into arm 3 to allow recruitment into arm 5 to catch up) it is now anticipated that the primary outcome measure for the original experimental arm 3 will be reached after 7.5 years. We will review assumptions at each interim stage and present to the DMC.

The artpep program in Stata® will be used with real-time recruitment data to predict when these time points are likely to occur.

### Methods for additional analyses (e.g. subgroup analyses) {20b}

Pre-planned subgroup analyses of OS and EFS will include the stratification factors (e.g. OPC risk), any differences in methodology, e.g. three weekly versus weekly cisplatin, the two permitted versions of radiotherapy planning, and other key prognostic factors. It is expected that good correlation might be seen between p16 and HPV results. Several papers support the idea that there might be a better treatment effect with less radiation of tissue in the 5 + 5 group of the radiotherapy outlining technique, and in this trial, it is anticipated that if there is a difference in the subgroup analysis, it might be more promising in the 5 + 5 group.

Prespecified subgroup analysis will also be performed on OS and EFS for the arm 1 versus 5 comparison by PD-L1 expression status from translational analyses using both the TPS and CPS scoring methods using multiple predefined cut-points for each method.

### Methods in analysis to handle protocol non-adherence and any statistical methods to handle missing data {20c}

For the analysis of survival outcomes for patients that do not experience an event, they will be censored at the date that they were last known to be event-free. For the QoL outcomes, if there are substantial missing data, then various strategies will be employed as sensitivity analyses to evaluate the impact on outcomes (for example, different methods of analysis, interpolation between time points and multiple imputation). For the EQ-5D questionnaire, if a patient has died, then a score of 0 will be used as their utility score for the time points that they have not completed.

### Plans to give access to the full protocol, participant-level data, and statistical code {31c}

Participant data and the associated supporting documentation will be available within 6 months after the main trial manuscript is published. Details of our data request process are available on the CRCTU website. Only scientifically sound proposals from appropriately qualified research groups will be considered for data sharing. The decision to release data will be made by the CRCTU Director’s Committee, who will consider the scientific validity of the request, the qualifications and resources of the research group, the views of the chief investigator and the trial management and steering committees, the consent arrangements, and the practicality of anonymising the requested data and contractual obligations. A data sharing agreement will cover the terms and conditions of the release of trial data and will include publication requirements, authorship, and acknowledgements and obligations for the responsible use of data. An anonymised encrypted dataset will be transferred directly using a secure method and in accordance with the University of Birmingham’s Information Technology Services guidance on the encryption of datasets.

### Oversight and monitoring

#### Composition of the coordinating centre and trial steering committee {5d}

The trial was set up and is being managed and analysed in the UK by the CRCTU at the University of Birmingham on behalf of the sponsor (University of Birmingham) according to its standard policy and procedures. The TMG is composed of the chief investigator, clinical co-ordinators, co-investigators, invited principal investigators, lead and trial statistician(s), senior trial manager, and trial coordinator. The TMG is responsible for the day-to-day running and management of the trial and meets regularly usually via teleconference.

The TSC provides overall supervision for the trial and ensures it is being conducted in accordance with the principles of GCP. Membership will be composed of the TMG, invited principal investigators, representatives from the funders, network manager, a patient and public involvement representative, and an independent chair. The TSC will meet shortly before the commencement of the trial and then annually. The TSC remit will include monitoring trial progress including recruitment, data completeness, protocol compliance, and review of updated information. They will make recommendations about the conduct and continuation of the trial and whether interim data may be published.

#### Composition of the data monitoring committee, its role, and reporting structure {21a}

Data analyses will be supplied in confidence to an independent DMC, which will be asked to give advice on whether the accumulated data from the trial, together with the results from other relevant research, justifies the continuing recruitment of further patients. The DMC will operate in accordance with a trial-specific charter based on the template created by the Damocles Group. The DMC will meet 6 months after the commencement of recruitment and then annually during the recruitment phase to toxicity and review safety data for each treatment arm and findings from the interim analysis. Analyses will only be conducted once the required number of control events has been observed for each comparison. Additional meetings may be called if recruitment is much faster than anticipated and the DMC may, at their discretion, request to meet more frequently or continue to meet following completion of recruitment. An emergency meeting may also be convened if a safety issue is identified.

The DMC will report directly to the TMG who will convey the findings of the DMC to the TSC. The DMC may consider recommending the discontinuation of the trial if the recruitment rate or data quality are unacceptable or if any issues are identified which may compromise patient safety. The DMC can recommend premature closure or reporting of the trial or that recruitment to any research arm be discontinued. The DMC can also decide whether interim data may be published.

### Adverse event reporting and harms {22}

The collection and reporting of adverse events (AEs) is undertaken in accordance with the Medicines for Human Use Clinical Trials Regulations 2004 and its subsequent amendments. Definitions of different types of AEs are listed in Additional file 1: Appendix 7.

AEs are commonly encountered in patients receiving chemoradiotherapy, with the safety profiles of the investigational medicinal products (IMPs) used in this trial well-characterised. Therefore, the focus of data collection is AEs likely to be related to the trial treatments being studied (i.e. adverse reactions (ARs)/toxicities). Investigators will report AEs that meet the definition of a serious adverse event (SAE) as part of the SAE Completion Guidelines. The investigator will assess the seriousness and causality (relatedness) of all AEs experienced by the patient (this should be documented in the source data) with reference to the Summary of Product Characteristics for the IMPs used in the study. The principal investigator is responsible for ensuring that all the staff involved in the study are familiar with AEs and their reporting.

#### Durvalumab SAE reporting

Durvalumab adverse events of special interest (AESIs) specific to the use of durvalumab treatment in arm 5 will be reported at baseline and during treatment. Guidelines for the management of immune-mediated reactions, infusion-related reactions, and non-immune-mediated reactions for durvalumab are provided in Additional file 1: Appendix 4. Patients will be thoroughly evaluated, and appropriate efforts should be made to rule out neoplastic, infectious, metabolic, toxin, or other etiologic causes of the immune-mediated adverse event (imAE). Serologic, immunologic, and histologic (biopsy) data, as appropriate, should be used to support an imAE diagnosis. In the absence of a clear alternative aetiology, events should be considered potentially immune-related. In certain circumstances, durvalumab may be permanently discontinued.

### Frequency and plans for auditing trial conduct {23}

On-site and remote monitoring will be carried out as required following a risk assessment, as documented in the national monitoring plans, and as documented in the CompARE Quality Management Plan. Additional on-site monitoring visits may be triggered for example by poor CRF return, poor data quality, low SAE reporting rates, and excessive number of patient withdrawals or deviations.

### Plans for communicating important protocol amendments to relevant parties (e.g. trial participants, ethical committees) {25}

Protocol modifications will be notified to the competent authority, ethics committee, and investigators by the CompARE Trial Office. Where relevant, for instance when an arm is closed to recruitment, specific notification will be sent to all patients who may be affected, with a discussion of the findings that resulted in closure of the arm to recruitment and a list of possible treatment options.

### Dissemination plans {31a}

The primary routes for dissemination of the results of the trial will be, for healthcare professions, via conference presentations and publication in peer-reviewed journals and via national and international specialty associations, e.g. IFHNOS, HNCIG, and BAHNO. For participants and the public, dissemination will be via websites of relevant cancer charities and other organisations such as Cancer Research UK and ClinicalTrials.gov and via patient organisations, e.g. The Swallows, Oracle, and HANCUK.

## Discussion

### Trial design

An open-label randomised controlled trial using a MAMS design has been utilised for this study as it allows several treatment regimens to be assessed simultaneously against a single control arm. It is designed to be efficient and cost-effective as it allows through interim analysis a research arm to be discontinued if it appears not to be effective. In addition, the adaptive MAMS design enables flexibility in response to new data, emerging new treatments or difficulties in recruitment as it allows continuing recruitment to be focused on treatment regimens that show promise, whilst discontinuing investigation of regimens with insufficient evidence of activity. To date, the main changes to the protocol have been:

- Suspension of recruitment to experimental arms 2, 3, and 4
- Addition of arm 5 (immunotherapy)
- Changes to eligibility criteria with the addition of HPV N3 and T4 to the eligibility criteria as an intermediate-risk group
- A change to the randomisation ratio for arm 5, from 2:1 to 1:1
- Adding a new outlining radiotherapy protocol to enable 5 + 5 outlining

### Suspension of recruitment to arm 3

Recruitment to arm 3 was suspended in advance of the planned interim analyses due to a SAE resulting in death. Results are currently being prepared for publication [19], with harms and quality of life results of the dose-escalated chemoradiation arm [20]. A DMC investigation was undertaken to explore causality, and this did not find any causal relationship between the event and the study intervention. For further surety, the trial management team took the decision to await the interim data analysis prior to further recruitment. Results are still awaited.

### Outcome of QRI

Recruitment of patients into the QRI was initiated with a trial opening on 6 July 2015 and was completed on 12 December 2018. The aim of the QRI was to characterise and understand the success/failure of the trial recruitment process. Phase I included understanding the patient pathway through eligibility and recruitment; in-depth, semi-structured interviews with members of the TMG, clinical and recruitment staff, and participants eligible for recruitment to the trial; and audio-recording of investigator meetings and recruitment appointments. In phase II, the QRI team presented a summary of anonymised data from phase I to the TMG.

## Trial status

The study opened to recruitment on 6 July 2015 and is currently on protocol version 8.0b (2nd June 2020). Arm 2 closed to recruitment on 9 January 2017, arm 3 on 12 September 2019, and arm 4 on 7 February 2019. Recruitment is currently open to the control arm (arm 1) and arm 5, induction durvalumab plus arm 1 followed by adjuvant durvalumab. Recruitment to CompARE is expected to continue until at least January 2024.

### Supplementary Information

**Additional file 1: Appendix 1.** CompARE study sites. Lists of those trial sites that are actively recruiting, have paused recruitments, and have closed to recruitment. **Appendix 2.** Exemplar CompARE patient information sheets. Current patient information sheets for arms 1 and 5, as well as the trial summary information sheet. **Appendix 3.** Exemplar CompARE informed consent forms. Current informed consent forms for arms 1 and 5, as well as for the optional sub-study, CompARE Collect. **Appendix 4.** Dose modification and toxicity management guidelines for immune-related, infusion-related, and non-immune-mediate reactions for durvalumab. Guidelines for dose modification and toxicity management for durvalumab (arm 5) are listed. **Appendix 5.** Prohibited concomitant medications for use during cisplatin therapy (arm 1). A list of those medications prohibited for patients in arm 1. **Appendix 6.** Prohibited concomitant medications for use with durvalumab (arm 5). A list of those medications prohibited for patients in arm 5. **Appendix 7.** Definitions of adverse events. Definitions of adverse events used during the CompARE trial are included.

## Data Availability

No data are presented in this manuscript. The materials described can be obtained by contacting the corresponding author.

## Abbreviations

AE: Adverse event
AESI: Adverse events of special interest
AR: Adverse reaction
CRCTU: Cancer Research UK Clinical Trials Unit
CRF: Case report form
CTCAE: Common Terminology Criteria for Adverse Events
DMC: Data monitoring committee
EFS: Event-free survival time
EORTC: European Organisation for Research and Treatment of Cancer
EQ-5D: EuroQol Group
eRDC: Remote Data Capture
F: Fractions
GCP: Good Clinical Practice
HPV: Human papillomavirus
HR: Hazard ratio
IIT: Intention-to-treat
imAE: Immune-mediated adverse event
IMP: Investigational medicinal product
IMRT: Intensity-modulated radiotherapy
IV: Intravenous
LRC: Loco-regional control
MAMS: Multi-arm multi-stage
MDADI: M.D. Anderson Dysphagia Inventory
MDT: Multidisciplinary team
ONS: Office of National Statistics
OPC: Oropharyngeal cancer
OS: Overall survival time
PCR: Polymerase chain reaction
PED: Percutaneous endoscopic gastrostomy
QoL: Quality of life
QRI: QuinteT Recruitment Intervention
RTOG: Radiation Therapy Oncology Group
SAE: Serious adverse event
TMG: Trial Management Group
TNO: Trial number
TSC: Trial Steering Committee
