# Supplementary material for: CompARE: study protocol for a phase III randomised controlled platform trial comparing alternative regimens for escalating treatment of intermediate and high-risk oropharyngeal cancer
Source: Trials. 2024 Jan 15;25:50. doi: 10.1186/s13063-023-07881-1 (PMC10788973; doi:10.1186/s13063-023-07881-1)
Supplement: Supplementary file 1 — Additional file 1: Appendix 1. CompARE study sites. Lists of those trial sites that are actively recruiting, have paused recruitments, and have closed to recruitment. Appendix 2. Exemplar CompARE patient information sheets. Current patient information sheets for arms 1 and 5, as well as the trial summary information sheet. Appendix 3. Exemplar CompARE informed consent forms. Current informed consent forms for arms 1 and 5, as well as for the optional sub-study, CompARE Collect. Appendix 4. Dose modification and toxicity management guidelines for immune-related, infusion-related, and non-immune-mediate reactions for durvalumab. Guidelines for dose modification and toxicity management for durvalumab (arm 5) are listed. Appendix 5. Prohibited concomitant medications for use during cisplatin therapy (arm 1). A list of those medications prohibited for patients in arm 1. Appendix 6. Prohibited concomitant medications for use with durvalumab (arm 5). A list of those medications prohibited for patients in arm 5. Appendix 7. Definitions of adverse events. Definitions of adverse events used during the CompARE trial are included. [file 13063_2023_7881_MOESM1_ESM.pdf]

# Supplementary Appendix 1: CompARE study sites

| **Actively recruiting:** |
|---|
| Queen Elizabeth Hospital, Birmingham |
| Norfolk & Norwich University Hospital |
| Weston Park Hospital, Sheffield |
| James Cook University Hospital |
| Aberdeen Royal Infirmary |
| Royal Preston Hospital |
| St James's Hospital, Leeds |
| Bradford Royal Infirmary |
| Bristol Haematology and Oncology Centre |
| Oxford Cancer and Haematology Centre |
| North Middlesex Hospital |
| Colchester General Hospital |
| Western General, Edinburgh |
| Queen's Hospital Romford |
| Singleton Hospital, Swansea |
| Clatterbridge Cancer Centre |
| Derriford Hospital, Plymouth |
| Musgrove Park Hospital, Taunton |
| Torbay Hospital |
| Addenbrooke's Hospital, Cambridge |
| Royal Shrewsbury Hospital |
| The Christie Hospital |
| Belfast City Hospital |
| Cheltenham General Hospital |
| The Beatson, Glasgow |
| Royal United Hospital, Bath |

| |
|---|
| St Luke’s Hospital & St James’s Hospital, Dublin |
| University Hospital Galway |

| **Paused recruitment:** |
|---|
| Royal Devon & Exeter Hospital |

| **Closed to recruitment:** |
|---|
| *York Hospital* |
| *Velindre Cancer Centre, Cardiff* |
| *Castle Hill Hospital* |
| *Freeman Hospital, Newcastle* |
| *University Hospital Coventry (recruitment suspended - do not offer arm 5)* |
| *Newcross Hospital (recruitment suspended - do not offer arm 5)* |
| *Nottingham City Hospital* |
| *Leicester Royal Infirmary* |

To be printed on hospital headed paper

# Patient Information Sheet E
# (Arms 1 and 5)

**Phase III randomised controlled trial Comparing Alternative Regimens for escalating treatment of intermediate and high-risk oropharyngeal cancer**

EudraCT No.: 2014-003389-26
IRAS No.: 161147

We would like to invite you to take part in a non-commercial clinical trial (research study) called CompARE. The research study is run by the Cancer Research UK Clinical Trials Unit, University of Birmingham. It is funded by Cancer Research UK and sponsored by the University of Birmingham. Before you decide if you would like to take part, we would like you to understand why the research is being done and what it would involve for you. Please take time to read the following information carefully and discuss it with friends and relatives if you wish. Your study doctor will go through this information sheet with you and answer any questions you may have. This information leaflet is divided into two parts:

- Part 1 explains the purpose of the study and what will happen to you if you decide to take part.
- Part 2 provides you with more detailed information about the conduct of the study.

Please tell your study doctor or research nurse if there is anything that is not clear or if you would like more information. Take your time to decide whether or not you want to take part. If you decide not to take part, this will not affect your standard of care.

## Part 1

### What is Oropharyngeal Cancer?

Oropharyngeal cancer is a disease in which malignant cells form in the tissue of the oropharynx. The oropharynx is a middle part of the throat which includes the base of the tongue, the tonsils, the soft palate, the uvula, and the walls of the throat. Oropharyngeal cancer is usually treated with cisplatin chemotherapy and radiotherapy (chemoradiotherapy), which is considered the standard treatment in the UK.

The main causes of oropharyngeal cancer are:

1. Tobacco smoking or drinking alcohol
2. Human Papillomavirus: There are over 100 types of Human Papillomavirus and infection with Human Papillomavirus is very common. It is usually passed by skin-to-skin contact or sexual intercourse. Many people will often catch a Human Papillomavirus infection in early adulthood, and approximately 80% of women will have had at least one infection by the age of 28. Most Human Papillomavirus infections cause no harm or may cause warts on the skin. A small group of Human Papillomavirus infections, called high-risk Human Papillomaviruses, can cause cancer such as cervical cancer or throat cancer. But even then, most people infected with high-risk Human Papillomavirus will not have any diseases or cancer and will not suffer any harm. Only a very small number of patients will develop cancers, can be 20-30 years after infection.

Oropharyngeal cancer can be divided into two types:
**Low-risk oropharyngeal cancer:** about one third of oropharyngeal cancers are classified as low-risk. These are patients whose cancers are caused by the Human Papillomavirus and who do not smoke or smoke very little. These low-risk oropharyngeal cancers appear to respond very well to chemoradiotherapy which has a cure rate of more than 90%.

**Higher-risk (includes high-risk and intermediate-risk) oropharyngeal cancer:** the other two thirds of oropharyngeal cancers are classified as higher-risk. These are patients whose cancers are caused mainly by heavy tobacco smoking or alcohol intake. About half of these higher-risk cancers also have Human Papillomavirus in them. These higher-risk oropharyngeal cancers respond less well to the chemoradiotherapy, with cure rates of about 50-70%.

**The CompARE study is only for patients who have higher-risk oropharyngeal cancer which can be more resistant to standard treatment. Your doctor thinks you may benefit from taking part in this study.**

### What is the purpose of the study?

The CompARE study will help us decide which treatment regimen is most effective for treating patients who have higher-risk oropharyngeal cancer. To answer this question, the study compares two treatments. The treatment regimen uses an additional treatment in addition to standard chemoradiotherapy to try to increase cure rates of higher-risk oropharyngeal cancer. This will be done by adding immunotherapy to the standard chemoradiotherapy. The different treatments are described in more detail later in the patient information sheet.

This Patient Information Sheet covers two different treatment regimens in the CompARE study. Your study doctor will discuss treatment options with you.

The study also has additional parts:

- **Quality of Life**: This will help us find out about how your treatment might affect your Quality of Life and well-being. We think it is very important to find out how patients taking part in the CompARE study feel, both emotionally and physically and to study any side effects in some detail. The information will be used to compare advantages and disadvantages of each treatment combination. Your study nurse will ask you to complete various questionnaires in clinic; this is explained in more detail later in this information sheet.

- **CompARE Collect - Optional**: This will allow scientists to study blood, tumour and oral fluid samples from patients with higher-risk oropharyngeal cancer so that they can try to understand how the cancer cells work and to study the effects of the drugs used in the trial. In particular we will try to identify 'markers' that may predict which treatments will work for which patients to help select the most effective treatment for each patient.

- We will also use the samples to understand why cancers develop to help us to find new treatments for them. We also want to look at how genes in the cancer are changed and how the immune system works to get rid of the tumour. Also scientists may want to study your samples using new advanced research techniques that become available in future to help them understand more about cancer and how to diagnose and treat it. You will be asked if you want to provide blood and oral fluid samples as well as tissue from your diagnostic biopsy and pieces of any tissue removed surgically that are no longer needed. If you agree you will be asked to sign the CompARE Collect Informed Consent Form.

### *What treatments are being tested?*

We are testing two different types of treatment regimens both of which contain the standard treatment of chemoradiotherapy. Radiotherapy will be delivered using a method known as Intensity Modulated Radiotherapy which increases the radiotherapy dose delivered to the resistant cancer. Your clinical team will have assessed you as being eligible for the following treatments offered at your centre:

- **Group 1 (standard treatment):** Chemoradiotherapy (a drug called cisplatin (or carboplatin in some cases) + radiotherapy)

- **Group 5:** Induction Immunotherapy (durvalumab) + chemoradiotherapy (as per Group 1) + adjuvant immunotherapy (durvalumab)

### What is Cisplatin?

Cisplatin is an anti-cancer drug which prevents cancer cells from dividing and kills them. It is the standard drug used to treat head and neck cancers.

### What is Carboplatin?

Carboplatin is an anti-cancer drug for the treatment and management of head and neck cancers. It works by interfering with DNA affecting cell division and cell growth.

### What is Durvalumab?

Durvalumab is an investigational immunotherapy drug that has not yet been licensed for the treatment of head and neck cancer, but is in development for the treatment of a variety of different types of cancer. It is manufactured and supplied by the company AstraZeneca.

Durvalumab is an antibody designed to boost the body's own immune system by targeting a protein on tumour cells called programmed cell death ligand 1 (PDL-1). It is one of a group of compounds that inhibit PD-L1 that

have been widely used in lung cancer, and have shown impressive results in other types of cancer. In cancer, PDL-1 helps tumours evade detection and elimination by the cells of the immune system. Durvalumab blocks this effect and allows the immune cells to identify and destroy cancer cells.

## What is Intensity Modulated Radiotherapy?

Radiotherapy stops cancer cells from dividing and growing, thus slowing or stopping tumor growth. Intensity Modulated Radiotherapy is an advanced type of radiation which allows us to give high doses of radiotherapy to the tumour and at the same time reducing the dose to healthy tissue to reduce side effects. It is now a common standard practice for head and neck cancers.

## Why have I been invited to take part?

You have been invited to take part in this study because you have been diagnosed with oropharyngeal cancer that we believe is 'higher-risk' and the standard treatment for you would be chemoradiotherapy. All suitable patients are being approached to see if they would like to take part in this study. 695 patients with higher-risk oropharyngeal cancer will be invited to take part in this study around the UK.

## Do I have to take part?

It is up to you to decide whether or not to take part in this study. Your participation is entirely voluntary. You may choose not to participate. If you decide to take part you are free to withdraw at any time without having to give any reason for your decision. This will not affect the standard of care you receive.

## What will happen to me if I take part?

### *Before starting the study:*

**Initial consultation:** During the initial consultation, your study doctor will have given you a Trial Summary Sheet and explained to you the procedures for Human Papillomavirus testing as part of eligibility screening. If you decided to take part in the initial investigations, you would have been asked to sign Informed Consent Form A (Registration). Once you had done this, your study doctor registered your intent to take part in the study with the CompARE Trial Office at the Cancer Research UK Clinical Trials Unit, University of Birmingham and assigned a unique Trial Number. Screening tests would have been performed to check if you were suitable to take part in the study.

**Second consultation:** If screening shows that you are eligible, you will be asked whether you are interested in taking part in the CompARE study and will be provided with more information about the study. Your study doctor or research nurse will explain the aims, trial treatment, anticipated benefits and potential hazards of taking part in this study. You will be given a copy of the Patient Information Sheet to keep for your records and given time to discuss with your family, friends and also your GP if you wish to take part in the study. If you decide to take part in the CompARE study you will be asked to sign Informed Consent Form D (Randomisation) to show that you have agreed to take part in the study. If you also agree to provide tissue, blood and oral fluid samples as part of CompARE Collect you will be asked to sign the CompARE Collect Informed Consent Form. You can still take part in the study even if you do not want us to collect your blood, oral fluid and tissue samples.

### *Screening Assessments:*

Sections of your diagnostic biopsy that was taken to show you have oropharyngeal cancer will have been sent to the Central Laboratory Services based at Newcastle Hospitals NHS Foundation Trust. Tests for Human Papillomavirus will be performed to check if the tumour is Human Papillomavirus related. The results of the Human Papillomavirus status can take up to a week. During this period other screening assessments will be

performed. If the central pathology review of your biopsy shows you have higher-risk oropharyngeal cancer, you will be suitable to take part in the CompARE study.

There is a small chance that it may not be possible to measure the Human Papillomavirus status on your biopsy. If this is the case, you will not be able to enter the study. If this happens your study doctor will discuss other treatment options with you.

After Human Papillomavirus testing, the diagnostic biopsy tissue samples will be sent to the University of Birmingham for future research if you have consented to take part in CompARE Collect. If you have not given consent for sample collection as part of CompARE Collect, the diagnostic biopsy tissue samples will be returned to your hospital.

Some additional tests will be performed as part of the screening assessments to ensure that it is appropriate and safe for you to participate in this study. These are summarised in table 1. You will also be asked questions about your health (e.g. smoking status, details of previous treatment or any other illness you may have) and what medicines you are taking. You should inform your study doctor if you have had any recent vaccinations or if you plan to receive any vaccinations. It is important to let your study doctor or research nurse know if you have diabetes, hypertension or suffer from any other illnesses. It is also important to tell your study doctor of previous and current tobacco and alcohol consumption. Your study doctor will decide if it is appropriate and safe for you to take part in this study.

A number of assessments will be performed as part of standard care as summarised in table 1. These include a full physical examination (measure of your height and weight), vital signs and performance status (measure of your general health and how your disease affects your daily routine), hearing test (not mandatory), dental assessments, swallowing assessments. In addition you may have a Computerised Tomography scan (also known as CT scan) or a Magnetic Resonance Imaging scan (also known as MRI scan) or a Positron Emission Tomography–Computed Tomography scan (also known as PET-CT scan). These scans will tell us the locations and size of the tumour(s). If necessary, you may also need to have a feeding tube known as a Percutaneous Endoscopic Gastrostomy (PEG) inserted. Each of these assessments is part of routine care and will be discussed with you. Some further laboratory tests will also be performed, including full blood count, renal function tests, liver function tests, Thyroid Stimulating Test (TSH), a pregnancy test if you are female and of child-bearing age (urine test or blood test),and tests for hepatitis B and C and Human Immunodeficiency Virus (HIV).

If you will be receiving durvalumab (Group 5), the research team will perform some additional laboratory tests (including blood and urine analysis) and electrocardiogram (ECG), which will record the electrical activity of the heart.

**Table 1: Screening Assessments**

| Assessments | Screening | Trial Entry Visit (at randomisation) | Additional baseline tests if allocated to Group 5 |
|---|---|---|---|
| Review of medical history* | x | | |
| Physical examination* | x | | |
| Hearing test** | x | | |
| Dental Assessment* | x | | |
| Swallowing Assessment * | x | | |
| CT or MRI or PET-CT scan* | x | | |
| Full blood count* | x | | |
| Thyroid Stimulating Test (TSH) | x | | |
| Pregnancy test# | x | | |
| Hepatitis B and C | x | | |
| Human Immunodeficiency Virus (HIV) | x | | |
| Obtain informed consent and Randomisation | | x | |
| Blood and Oral Fluid Collection for CompARE Collect*** | | x | |
| Completion of Questionnaires | | x | |
| Additional laboratory tests | | | x |
| ECG | | | x |

Keys:
*Assessment part of standard practice
**Hearing test is recommended, but not mandatory
***Samples collected only if you have consented to take part in CompARE Collect
# If you are female and of child-bearing age (urine test or blood test)
^Additional laboratory test: amylase, lactate dehydrogenase (LDH), aspartate aminotransferase (AST), pregnancy test, urine test
CT: Computerised Tomography scan; MRI: Magnetic Resonance Imaging scan; PET-CT: Positron Emission Tomography–Computed Tomography scan; ECG: Electrocardiogram

### *Study Entry:*

For patients where the Human Papillomavirus test shows their tumour is higher-risk, your study doctor will again call the CompARE Trial Office and you will be allocated a treatment. The treatment will be allocated through a process called randomisation. This means the doctors and you cannot select your treatment, it will be allocated at random by a computer based at the Cancer Research UK Clinical Trials Unit. This study is called an "open-label trial", which means that both you and your study doctor will know which treatment you will receive.

On the same day of study entry, you will be asked to complete various questionnaires in clinic. Your study doctor or research nurse will explain to you how to complete these questionnaires:

- Quality of Life Questionnaires: The questionnaires will ask about symptoms you are experiencing and how you are feeling.
- Swallowing Assessment Questionnaire: The questionnaire will ask about your swallowing ability and how you feel about swallowing.
- Cost Effectiveness Questionnaire: The questionnaire will ask about whether the disease and its treatment affect your finances (e.g. whether there is more cost because of additional hospital visits). This is to monitor and identify any financial burden to patients and their families caused by either of the treatments given in this study.

You will be issued a Patient Identification Card on which your patient Trial Number and allocated treatment will be recorded. Contact numbers if you feel unwell at any time are also recorded on the Patient Identification Card. You must keep the Patient Identification Card with you at all times.

If you have agreed and consented to take part in CompARE Collect, you will be required to provide a sample of your blood (approximately 40mls, equal to eight teaspoons) and oral fluid (a spit from the mouth). Samples will be sent to the University of Birmingham for storage for research tests to be done now and in future.

## *Study Treatment:*

You will start the allocated study treatment as soon as practical. You will receive chemoradiotherapy whichever group you are allocated to. The two treatment regimens are summarised in table 2.

**Chemotherapy:**
We usually describe chemotherapy treatment as being given in cycles of treatment. You will receive chemotherapy through a vein in your arm called infusion. The chemotherapy drug you will receive is called cisplatin. If your study doctor is concerned about your ability to tolerate cisplatin chemotherapy (for example, reduced kidney function) you will be given an alternative chemotherapy drug known as carboplatin, which is consistent with standard practice. Chemotherapy is a standard therapy and the side effects of cisplatin and carboplatin will be discussed later.

**Intensity Modulated Radiotherapy:**
**Treatment planning:** Radiotherapy has to be carefully planned to make sure it works as well as possible. You will usually have a Computerised Tomography scan and often a Magnetic Resonance Imaging scan to take 3D pictures of the area to be treated. This helps doctors and radiographers identify the exact shape and location of the tumour to be targeted by the radiotherapy. Treatment planning is very important part of radiotherapy, and it may take a couple of visits.

During the treatment preparation session you will have a mask made for you. You will be asked to wear the mask while you're having the treatment. This helps keep your head in the same position while the radiotherapy is being given. It also helps to avoid giving radiotherapy to parts of the head and neck that do not need treatment. The mask is made in the mould room of the radiotherapy department by a mould technician or radiographer. The process takes about 30-60 minutes. One technique uses wet plaster bandages to take an impression of your face, with the finished mask being made of perspex. The other technique uses a type of mesh plastic, which is moulded to fit the shape of your face.

**Treatment sessions:** Treatment with radiotherapy does not hurt and you cannot feel it. You are left alone in the room during your treatment but the radiographers are always watching you on cameras. Radiotherapy will be given 5 days a week for 7 weeks. Radiotherapy related side effects will be discussed later.

Cancer cells can spread from the site of the tumour, usually going to the neck glands. At 3 months, tumour response to treatment will be assessed, whichever group you have been allocated to. If the scans show that there is still cancer in the neck glands, then you will need a neck dissection. A neck dissection is an operation to remove some or all of the lymph glands in one or both sides of your neck. Your clinician will discuss this further with you if you need it.

**Immunotherapy Drug (Durvalumab):**
If you are allocated to Group 5, you will receive an initial dose of durvalumab called induction durvalumab followed by chemoradiotherapy. The induction durvalumab will be a fixed dose of 1500mg.This will be administered as a 1 hour intravenous infusion.

Following chemoradiotherapy you will receive adjuvant durvalumab treatment. You will be given durvalumab 1500mg as a 1 hour intravenous infusion every 4 weeks for 6 months. Side effects of durvalumab will be discussed later.

**Table 2: Treatment Options**

| Group 1: Chemoradiotherapy | |
|---|---|
| **Radiotherapy** | Delivered 5 days a week for 7 weeks |
| **Chemotherapy** | Cisplatin will be given via a thin tube through a vein in your arm either:<br>• Once every three weeks for three cycles (given on days 1, 22 and 43 from start of radiotherapy) usually as a 10 hour infusion during inpatient clinic and may require an overnight stay.<br>OR<br>• Once a week for 7 weeks (given on days 1, 8, 15, 22, 29, 36 and 43 from start of radiotherapy) usually as a 3 hour infusion during outpatient visit.<br><br>Extra medication and hydration therapy may also be given before or after chemoradiotherapy to reduce the risk of sickness and other side effects as per standard practice. |
| We will reassess the tumour 12 weeks after the end of the treatment and there may be the need to have an operation of the neck to remove any involved neck glands that did not respond to treatment. | |

| Group 5: Immunotherapy plus chemoradiotherapy | |
|---|---|
| **Induction Immunotherapy*** | First single dose of immunotherapy drug, durvalumab called induction durvalumab is delivered via 1 hour infusion. It is delivered via a thin tube through a vein in your arm. |
| **Radiotherapy** | As per Group 1, given after the end of the induction durvalumab. |
| **Chemotherapy** | As per Group 1, given after the end of the induction durvalumab. |
| **Adjuvant Immunotherapy**** | Durvalumab is delivered following chemoradiotherapy via 1 hour infusion. It is delivered via a thin tube through a vein in your arm and repeated every four weeks for 6 months. |
| We will reassess the tumour 12 weeks after the end of the chemoradiotherapy treatment and there may be the need to have an operation of the neck to remove any involved neck glands that did not respond to treatment. | |

*Definition of Induction: The first treatment given for a disease
**Definition of Adjuvant: Additional cancer treatment given after the primary treatment to enhance effectiveness of treatment or to lower the risk of cancer coming back

You will be monitored carefully during treatment. You will need to attend the clinic appointments at specified time points during treatment depending on the treatment you are receiving. The research team will perform routine laboratory tests (full blood count, renal function test, and liver function test) as well as some additional tests (i.e. if receiving durvalumab) at some or at all of the visits to see how the treatment is affecting your body. You will also be asked about any side effects you may have experienced and what medicines you are taking. You may also be required to complete additional questionnaires during your clinic appointments depending on the treatment you are receiving.

Once you have completed your treatment you will be invited back to clinic for end of treatment assessments. Laboratory tests as will be performed and you will be asked about any side effects you may have experienced. You will also be asked to complete some questionnaires in clinic. If you have consented to take part in CompARE Collect, blood and oral fluid samples will be collected at the end of chemoradiotherapy.

## Follow-up

After your treatment has finished you will have regular follow-up visits to check on your health. It is standard practice to follow you up closely initially and then to continue regular follow-up with reducing frequency for at least 2 years. You will need to attend clinic appointments monthly in the first year of follow-up and then 2 monthly in the second year. You will then be assessed annually for 5 years thereafter. The purpose of ongoing visits is

to monitor for relapse of the cancer. During the follow-up visits any side effects that may have occurred due to treatment will be recorded.

Some additional laboratory tests including a full blood count are planned during follow-up. You will also be asked to complete the questionnaires in clinic at 3, 6, 12, 18 and 24 months. A Positron Emission Tomography–Computed Tomography scan will be performed at the 3 month follow-up visit. If any persistent disease is identified in the neck, surgery will be performed. The assessments and tests performed during follow-up are summarised in table 3.

If you have consented to take part in CompARE Collect, any surplus tissue removed during surgery will be sent to the University of Birmingham and stored for future research. As part of CompARE Collect, you will also need to provide a sample of your blood and oral fluid at 3 and 12 months during follow-up. One extra blood and oral fluid sample may be requested at an additional time point during the follow-up period. Tissue, blood and oral fluid samples will be sent to the University of Birmingham.

**Table 3: Follow-up assessments**

| Assessments | Early Follow-up (year 1) | | | | | | | | | | | | Late follow-up (year 2) | | | | | | | | | | | | Annual follow-up | | | | |
|---|---|---|---|---|---|---|---|---|---|---|---|---|---|---|---|---|---|---|---|---|---|---|---|---|---|---|---|---|---|
| | 1 | 2 | 3 | 4 | 5 | 6 | 7 | 8 | 9 | 10 | 11 | 12 | 13 | 14 | 15 | 16 | 17 | 18 | 19 | 20 | 21 | 22 | 23 | 24 | 1 | 2 | 3 | 4 | 5 |
| PET-CT (if standard practice at your hospital)* | | | x | | | | | | | | | | | | | | | | | | | | | | | | | | |
| Full Blood Count* | | | x | | | x | | | | | | x | | | | | | x | | | | | | x | | | | | |
| Blood and Oral Fluid Collection for CompARE Collect** | | | x | | | | | | | | | x | | | | | | | | | | | | | | | | | |
| Completion of Resource Use Questionnaire | | | x | | | x | | | | | | x | | | | | | x | | | | | | x | | | | | |
| Review of side effects | x | x | x | x | x | x | x | x | x | x | x | x | | x | | x | | x | | x | | x | | x | x | x | x | x | x |
| Sugery if required | | | x | | | | | | | | | | | | | | | | | | | | | | | | | | |

Keys:
* Assessment which is part of standard practice
** Samples collected if you have consented to take part in CompARE Collect (may also be requested at one additional time point during follow-up)
PET-CT: Positron Emission Tomography–Computed Tomography scan

## Expenses and payments

You will not receive any money for taking part in this research study and unfortunately we cannot reimburse travel and parking expenses. Transport services may be available at your local hospital. However you would need to discuss access to these with your study doctor.

## What will I have to do?

If you agree to take part in the study you will need to:

- Complete and sign the Informed Consent Form(s)
- Attend the hospital as requested for scheduled clinic visits and scans
- Tell your study doctor or research nurse about your diet and lifestyle (alcohol consumption and tobacco use)
- Tell your study doctor or research nurse about all the medicines you take or have recently taken including regular prescriptions and non-prescription medicines (including herbal medications, vaccinations and over-the-counter medications) before starting treatment. It is also important that you tell your study doctor or research nurse about any changes to your medication at any time, or any new medication that is prescribed during the study. Any prohibited medication must not be taken throughout the study, your study doctor will tell you about these prohibited medications
- Tell your study doctor or research nurse if you have experienced a previous reaction to dyes used during a Computerised Tomography scan
- Tell your study doctor or research nurse about any symptoms or side effects you experience
- Complete questionnaires at defined time points during some clinic appointments

- Carry the Patient Identification Card that we will provide, that indicates you are taking part in this study
- Attend follow-up visits after your treatment

If you receive durvalumab as part of your treatment, you must agree to the following:

**Female patients (from the time of giving consent to 6 months following the last dose of durvalumab):**

- Avoid pregnancy unless you are unable to become pregnant (e.g. have had your "tubes tied", had a hysterectomy or are at least 1-year post-menopausal). If able to become pregnant use a highly effective method of birth control. Your study doctor will discuss contraceptive options with you.
- Refrain from breastfeeding and egg cell donation.
- Refrain from blood donation,

**Male patients (with a female partner from the time of giving consent to 6 months following the last dose of durvalumab):**

- Use a highly effective method of birth control. Your study doctor will discuss options with you.
- Refrain from sperm donation.
- Refrain from blood donation.

## What are the alternative treatments?

If you decide not to take part in the study, you will receive standard treatment for oropharyngeal cancer. Your doctor will explain what this will involve.

## What are the possible disadvantages and risks of taking part?

Whilst on the study, you will be asked to complete questionnaires during some of your hospital visits; as a result these visits will take a little more time than usual.

Possible side effects associated with treatment delivery and tests:

**Blood sample collection:** This may cause small amount of bleeding and temporary discomfort or you may feel faint. If this happens please tell the person taking the blood so that they can make sure you lie down until you are feeling better. Sometimes a bruise or redness develops at the site where the needle was inserted (but this will clear after a week or two). Please inform your study doctor or research nurse if you experience any reactions at the injection site.

**Computerised Tomography scan:** Prior to a Computerised Tomography scan, a dye will be injected into one of your vein which helps identify specific organs and blood vessels and improves accuracy of the scan. When the dye is injected, you may feel a warm or burning sensation in the area where the dye is injected. You may experience nausea, flushing and a salty taste in the mouth. There is a 2 in 1000 risk of an allergic reaction to the dye. In most cases these reactions are mild (such as a skin rash). Severe reactions such as respiratory problems account for 1 in 2500 of cases.

You will need to lie on a bed in the scanner which passes through a ring that sends a beam of x-rays through your body and is used to create internal images. You must not move during the test, but relax and breathe normally. The process is painless; however you might be uncomfortable and feel claustrophobic when you are in the tunnel-shaped machine.

**Magnetic Resonance Imaging scan:** Prior to a Magnetic Resonance Imaging scan, a dye will be injected into one of your veins using a small needle or plastic tube. You may feel local warmth or pain in the area where the dye is injected. Side effects from the dye may include nausea, vomiting or headache. Allergic reactions are rare.

**Chemotherapy delivery:** As chemotherapy is injected into your vein, you may feel some discomfort or pain.

**Neck dissection:** You may have one or two thin, plastic drainage tubes coming from the operation area, with bottles attached to them to collect fluid from the wound. This helps the wound to heal. Drains usually stay in place for about 2-7 days. As with all surgery, there is a risk of bleeding, infection or wound breakdown. There is also a small risk of a collection of blood or fatty fluid (chyle) which may delay your recovery. Shoulder pain and movement disability can happen in some patients. Your ear may also feel numb. It's very important to let your study doctor or research nurse know as soon as possible if you have any pain.

**Durvalumab delivery:** Reactions may occur during or after the infusion of study medication. The reaction may cause fever or chills and a change in blood pressure or difficulty in breathing which might be serious. Tell your study doctor right away if you experience any of these symptoms even if it has been several days after the infusion has been completed.

## What are the side effects of the treatment received when taking part?

You may have side effects whilst on the study. All treatments can have side effects (known as adverse events or toxicities). You will be monitored carefully for any side effects which may be mild or very serious.

## Intensity Modulated Radiotherapy

**Early side effects:** Tiredness, pain and difficulty swallowing due to mouth ulceration, altered sense of taste, nausea, skin soreness, thickened secretions and hair loss to the area near to where the radiotherapy is given. All these are common side effects with standard radiotherapy to the head and neck. The symptoms are typically worst at the end of treatment and start to gradually improve a few weeks after treatment has finished. Due to swallowing difficulties there is also an increased risk of chest infection (pneumonia) during this period.

**Late side effects (more than 3 months after radiotherapy):** Skin and cosmetic changes to the area where the radiotherapy was given, dental decay, difficulty in swallowing, dry mouth and a hoarse voice may occur. Up to one in ten patients experience difficulty in feeding sometimes requiring a feeding tube temporarily. Uncommon side effects include brachial plexus damage (damage to the network of nerves that sends signals from your spine to your shoulder, arm and hand) and rarely spinal cord damage which could result in disability (less than 1 in 100 cases).

All the above side effects occur with standard radiotherapy treatment.

## Chemotherapy

Chemotherapy has several side effects that are well recognised. A common side effect from all chemotherapy drugs is a risk of infections due to low white blood cells. You may also develop high fever as a result of this. You must tell your study doctor or research nurse if you suffering from fever immediately. Other common side effects of chemotherapy are nausea, vomiting, weakness, tiredness, sore mouth and mouth ulcers, diarrhoea, anaemia (reduction in red blood cells) and loss of appetite.

## Side effects for Cisplatin and Carboplatin

| | Very common side effects (affects more than1 patient in every 10) | Common side effects (affects less than 1 in 10 but more than 1 in 100 patients) |
|---|---|---|
| **Cisplatin** | • Reduction in blood platelets, which increases the risk of bruising and bleeding<br>• Reduced level of sodium<br>• Increased levels of uric acid (a chemical created when the body breaks down certain substances) in your blood<br>• Slight loss of hearing | • Blood-poisoning (sepsis)<br>• Increased, decreased, erratic or irregular heartbeat<br>• Inflammation of a vein at injection site<br>• Difficulty in breathing and inflammation of the lungs (pneumonia) |
| **Carboplatin** | • Changes in your red and white blood cells and platelets | • Allergic reaction including rash, skin reddening, itching, high temperature |

| | | |
|---|---|---|
| | • Slight loss of hearing<br>• Anaemia (a condition in which there is a decreased number of red blood cells which lead to tiredness)<br>• Increase in the level of urea in your blood<br>• Abnormal liver enzyme levels<br>• Reduced levels of sodium, potassium, calcium and magnesium | • Pins and needles (peripheral neuropathy)<br>• Ringing in the ears (tinnitus) and hearing impairment<br>• Increased, decreased, erratic or irregular heartbeat<br>• Unusual bruising or bleeding<br>• Difficulty in breathing, wheezing<br>• Visual disturbances<br>• Increase in the level of the creatinine, uric acid (which may lead to gout) and bilirubin in your blood<br>• Musculoskeletal disorder (disorders of muscles, joints and tendons) |

The severity of these symptoms can vary from person to person. Anti-sickness medication may be given to prevent vomiting. If you do take part in this study, you must report all symptoms to your study doctor and research nurse. There are contact numbers given at the end of this information sheet and on your Patient Identification Card if you feel unwell during treatment.

## Durvalumab

Durvalumab works by boosting the immune system. This may cause side effects, which can occur when the drug is given or after the drug is given (within hours, days or weeks after). Some side effects usually get better without any treatment. However, some side effects may become serious.

Side effects that may be experienced as a result of the immune system reactions to durvalumab include but are not limited to the following:

| Very common side effects (affects more than 1 in 10 patients treated): | Common side effects (affects between 1 in 100 and 1 in 10 patients treated): | Uncommon side effects (affects between 1 in 1,000 and 1 in 100 patients treated) | Rare side effects (affects between 1 in 10,000 and 1 in 1,000 patients treated) |
|---|---|---|---|
| • Diarrhoea<br>• Rash/dry itchy skin<br>• Feeling tired<br>• Nausea<br>• Vomiting<br>• Abdominal pain<br>• Upper respiratory tract infections<br>• Decreased appetite<br>• Shortness of breath<br>• Cough/productive cough<br>• Fever | • Inflammation in the lungs<br>• Low thyroid function<br>• High thyroid function<br>• Kidney problems<br>• Liver problems<br>• Nervous system problems<br>• Infusion Related Reactions<br>• Inflammation of the intestine<br>• Accumulation of fluid causing swelling<br>• Swelling of skin or hives at infusion site<br>• A hoarse voice<br>• Painful urination<br>• Night sweats<br>• Pneumonia<br>• Oral thrush<br>• Dental and oral soft tissue infection<br>• Pain in muscles and joints<br>• Influenza | • Inflammation of the pancreas<br>• Inflammation of the kidneys<br>• Inflammation of the liver<br>• Allergic reactions (these can cause swelling of the face, lips and throat, breathing difficulties along with hives or nettle like rash)<br>• Problems with adrenal glands<br>• Inflammation or damage to the tissues in the lungs<br>• Inflammation of the muscles or associated tissues such as blood vessels that supply the muscles (symptoms can include muscle weakness and aches, feeling tired when standing or walking and muscle pain or soreness that does not resolve after a few weeks) | • Type 1 Diabetes Mellitus which may cause increased blood glucose levels (symptoms may include weight loss, increased urination, increased thirst and increased hunger<br>• Diabetes insipidus which cause increased urination and increased thirst<br>• Problems with the pituitary gland (symptoms may include headaches, thirstiness, and trouble seeing or double vision, leakage of breast milk or irregular periods in women)<br>• Inflammation of the heart muscle (symptoms can include chest pain, rapid or abnormal heart beat, shortness of breath and swelling of your legs)<br>• Inflammation of the membrane surrounding the heart<br>• Growths of tiny collections of inflammatory cells in different parts of the body<br>• Inflammation of the middle layer of the eye and other events involving the eye (e.g. inflammation of the cornea and optic nerves)<br>• Hardening and tightening of the skin and connective tissues and loss of skin colour<br>• Haematological events (e.g. breakdown of red blood cells and low levels of platelets)<br>• Rheumatological events (inflammatory disorders causing muscle pain and stiffness and autoimmune arthritis)<br>• Inflammation of the blood vessels (vasculitis)<br>• Non-infectious irritation and inflammation of the meninges, the membranes covering the brain and spinal cord<br>• Non-infectious encephalitis (inflammation of the brain) |

In addition to the possible risks identified in patients treated with durvalumab, other immune-mediated side effects are possible that have not been observed, and can result in inflammatory side effects in any organ or tissue.

Bleeding has been reported in up to 10% or greater in patients with head and neck cancer. Reports of bleeding, including fatal reports, have been received from head and neck cancer patients enrolled in AstraZeneca clinical trials with durvalumab as monotherapy or in combination. It is not yet known whether the risk of bleeding would be higher or lower with durvalumab treatment than with standard chemotherapy. Please talk to your study physician immediately if you experience any bleeding. Please also tell your study physician if you are taking any medications that might increase your risk of bleeding, including aspirin, blood thinners, or certain types of pain medications called NSAIDs. Your study physician may suggest that you stop taking these medications while you are on the study drug.

It is possible your cancer will not improve during the study or may even worsen. There may be other risks involved in taking durvalumab, including the possibility of reactions that have not been identified in studies done so far. There is always a risk involved in taking this medication but every precaution will be taken to ensure your safety. If you experience any side effects or any changes in general health (whether you think it is related to the study treatment or not), it is important you report them to the study doctor or research nurse so that you can receive the necessary treatment.

## Harm to the unborn child and effects on fertility

The effects of the study treatment during pregnancy are not fully known. Therefore, it is important for all patients participating in the study (male and female) to use adequate contraception during your participation in the study and for at least six months after receiving treatment. Acceptable forms of contraception are barrier methods (e.g. condoms) for males and hormonal methods (e.g. contraceptive pill, implant or Minera coil) for females. Please discuss this with your doctor or nurse if you would like further information. For female patients, it is strongly recommended that you do not breastfeed during study treatment or for two months after your last dose of treatment. If you or your partner becomes pregnant during the course of the study, you should inform your doctor immediately.

All chemotherapy study treatments are known to have genotoxic effects and may alter male fertility. Therefore, men are advised not to father a child during and up to 6 months after treatment and to seek advice on conservation of sperm prior to treatment.

**Durvalumab:**
The effects of durvalumab on an unborn child are not known. It is very important that you use highly effective methods of birth control to prevent pregnancy during this study. If you are a female, you must not be pregnant or breastfeeding, and must not become pregnant during the study and for up to 6 months after the last dose of durvalumab. If you are male, you should agree to avoid getting your partner pregnant and you agree not to donate sperm while you are in the study and for up to 6 months after the last dose durvalumab. Your study doctor will advise you and check that the methods you are using are acceptable for your particular circumstance.

If during the treatment period, or for up to 6 months after durvalumab has been stopped, you learn that you are pregnant or your female partner becomes pregnant, you must tell your study doctor immediately. During your study visits the study doctor may ask you for more information about any pregnancy to see if there are any effects of the study medication on unborn children. If you are male and report a pregnancy of your female partner, the study doctor will ask to speak to your partner, if she agrees to this.

## Radiation and the Ionising Radiation (Medical Exposure) Regulations

You will be exposed to radiation during the Computerised Tomography scans or Positron Emission Tomography–Computed Tomography scans in addition to the therapeutic radiation of the radiotherapy used to treat the cancer:

**Computerised Tomography scan or Positron Emission Tomography–Computed Tomography scan:**

You will have a Computerised Tomography scan (of your neck and chest) or a Positron Emission Tomography–Computed Tomography scan as part of your screening assessments and at 3 months after the end of treatment. You will also receive a Computerised Tomography scan and other imaging as part of your radiotherapy preparation and your treatment process. As part of your regular clinical treatment with radiotherapy, you will need a Computerised Tomography scan to check the position of the radiation beam. All of these scans and imaging will be no different to what you would have as standard treatment if you were not participating in the study with the possible exception of a repeat Computerised Tomography scan or Positron Emission Tomography–Computed Tomography scan when you enter the trial. The radiation dose from EACH the Computerised Tomography scan or Positron Emission Tomography–Computed Tomography scan (whether for diagnosis or for checking the radiation beam position during your treatment) is equivalent to less than the natural background radiation in the UK that you would receive in 7 years. The risk from radiation you will receive from these various procedures is very low compared to the dose of radiation you get as part of your treatment. The potential benefits to you may outweigh the risks from radiation.

## What are the possible benefits of taking part?

There is evidence that in general patients who participate in studies have better outcomes than those who do not. However, we cannot predict whether you will benefit directly from taking part in this study. It may be that the treatment you get increases your chance of cure. However, we cannot guarantee the study will help you but the information we get from this study will help future treatment of patients with high-risk oropharyngeal cancer.

## What happens when the trial stops?

When you have finished your treatment, you will continue to be followed up regularly for at least 2 years and then annually for up to 5 years. This will allow us to gain information on long term side effects.

If the study stops before the end of your treatment, you will be treated according to the usual procedures at your hospital. This is unlikely; however you will be able to discuss this with your doctor to decide on the best course of action. Your progress will be followed in the same way as discussed earlier.

## What if there is a problem?

We will address any complaints you have about the way you have been treated during the study or any possible harm you might have suffered. More detailed information is given about this in part 2.

## Will my taking part in the trial be kept confidential?

Yes. All the information about your participation in the study will be handled in confidence. The details are included in Part 2.

**This completes Part 1.**
**If the information in Part 1 has interested you and you are considering participation, please read the additional information in Part 2 before making any decision.**

## Part 2

### What if relevant new information becomes available?

Sometimes during the course of a study, new information becomes available about the treatments that are being studied. If this happens, your study doctor will tell you about it and discuss whether you should continue with the study. If you decide not to carry on, your study doctor will make arrangements for your care to continue. If you decide to continue in the study you will be asked to sign an updated Informed Consent Form. Sometimes your study doctor might consider it to be in your best interests to withdraw you from the study. He/she will explain the reasons and arrange for your care to continue.

If the study is stopped for any other reason, you will be told by your study doctor why and your continuing care will be arranged.

### What will happen if I don't want to carry on with the study?

You are free to withdraw from the study at any time. You do not have to give a reason and your future treatment will not be affected. Your study doctor will discuss your treatment with you and will offer you the most suitable treatment available. However, if you choose to withdraw, we would still like to use the information we have collected up until withdrawal. If you decide to withdraw from the study it will not affect your medical care in any way. We would however like to ask your permission for your hospital to continue to send information on your progress to the CompARE Trial Office. The CompARE Trial Office will obtain information about your progress from the national health registries, with your permission. You may withdraw from any data collection, although any information collected up until your withdrawal may still be used for study purposes.

If you withdraw from the study your doctor will advise you about your treatment.

### What if there is a problem?

***Complaints***

If you have a concern about any aspect of this study, you should contact your study doctor or research nurse in the first instance who will do their best to answer your questions. You can use the contact number at the end of this sheet.

If you remain unhappy and wish to complain formally, you can do this through the Patient Advise and Liaison Services (PALS) or the National Health Service (NHS) complaints procedure. In Northern Ireland this can be done through the Health and Social Care (Northern Ireland) complains procedure. Details can be obtained from your hospital.

***Harm***

In the event that something does go wrong and you are harmed during the study and this is due to someone's negligence then you may have grounds for legal action for compensation against the Sponsor of the trial (University of Birmingham) or the NHS Trust treating you but you may have to pay your legal costs. NHS Trust and Non-Trust Hospitals have a duty of care to patients treated, whether or not the patient is taking part in a study, and the normal NHS complaints mechanisms will still be available to you. The Sponsor of the trial does not hold insurance against claims for compensation for injury caused by participation in this study and they cannot offer any indemnity.

### Will my taking part in the trial be kept confidential?

All information collected about you for this study will be subject to the EU General Data Protection Regulation and to the Data Protection Act 2018 for health and social care research and will be kept strictly confidential. University of Birmingham is the Sponsor for this study based in the UK. We will be using information obtained directly from your medical records in order to undertake this study and will act as the Data Controller for the

study. This means that we are responsible for looking after your information and using it properly. University of Birmingham and the NHS will keep identifiable information about you for at least 25 years after the study has finished.

All information including personal data collected indirectly or directly by the Sponsor will be securely stored at the CompARE Trial Office within the Cancer Research UK Clinical Trials Unit on paper and electronically and will only be accessible by authorised personnel. The only people in the University of Birmingham who will have access to information that identifies you will be people who manage the study or audit the data collection process.You can read the University of Birmingham Privacy Notice via the following web link: https://www.birmingham.ac.uk/privacy/crctu

At University Hospitals Birmingham only, this study will be managed by the InHANSE Trials Team at the University of Birmingham where the team looking after you reside. For convenience they would like to be able to hold study files and documents at the university rather than in the hospital. This means that some of your identifiable information may be held by InHANSE at the trials office on the University of Birmingham site. This includes the consent form you will sign if you agree to take part in the study. This information will remain confidential at all times. Only the doctors and nurses working on the study or providing healthcare for you will have access to any information that might identify you. You can still be involved in the research study even if you do not want your identifying information to be transferred to the university, in which case the documents and data will be held in a secure place in the hospital and university-employed doctors and nurses will only be able to access the data in the hospital.

With your permission, your study doctor or research nurse will provide your initials, date of birth, hospital and NHS number (or Community Health Index (CHI) in Scotland) when they enter you into the study. Your study doctor will notify your GP that you intend to participate in the study. They will also send a copy of your signed Informed Consent Form with your full name recorded in the post to the CompARE Trial Office.

The NHS will use your name and contact details to contact you about the research study, and make sure that relevant information about the study is recorded for your care, and to oversee the quality of the study. Your study doctor or research nurse may also need to send a copy of your Informed Consent Form to other healthcare professionals (e.g. your GP or NHS pathologist) to prove that you have given consent to take part in the study before they will provide information or tumour samples.

In the CompARE Trial Office you will only be identified by your unique Trial Number, initials, date of birth and hospital number. In addition your Trial Number, initials and date of birth will be included on the diagnostic tissue samples sent to the Central Laboratory Services for Human Papillomavirus testing. If you have consented to take part in CompARE Collect, your Trial Number, initials and date of birth will be recorded on the blood, oral fluid and any surgical tissue samples sent to the University of Birmingham.

All information will be treated as strictly confidential and nothing that might identify you will be revealed to any third party other than those involved in the treatment or organisation of tissue, blood and oral fluid sample collection and transfer (e.g. staff at University of Birmingham; Department of Cellular Pathology at Royal Victoria Infirmary and Hospitals where your diagnostic tissue samples are currently held). They have the same duty of confidentiality to you as all other research study personnel.

A copy of the radiotherapy treatment plan made for you before your radiotherapy starts will be sent to the CompARE Quality Assurance Team at Mount Vernon Hospital, Northwood, Middlesex. This includes copies of your CT/MRI and treatment images associated with this plan. These will be monitored and stored by the Quality Assurance team.

It may be necessary to send information about you such as trial number and date of birth to the collaborating company AstraZeneca who is providing durvalumab. This is for your and others protection to track the safety of the trial treatment, and for licensing purposes. They have the same duty of confidentiality to you as all other research trial personnel. By taking part in the study you will be agreeing to allow members of the research staff at your hospital and from the CompARE Trial Office to look at your study records, which includes your medical records. It may also be necessary to allow the authorised personnel from government regulatory agencies (e.g. the Medicines and Healthcare products Regulatory Agency), the Sponsor and/or NHS bodies to have access to information about you. This is to ensure that the study is being conducted to the highest possible standards. If

you give permission, we will also register your details with NHS Digital. This will enable us to collect additional long-term information.

All individuals who have access to your information have a duty of confidentiality to you. Under no circumstances will you be identified in any way in any report, presentation or publication arising from this study.

If you choose to withdraw from the trial treatment, we would still like to collect relevant information about your health, as this will be invaluable to our research. If you have any objection to this please let your study doctor know.

You can withdraw your consent to our processing of your data at any time. Your rights to access change or move your information are limited, as we need to manage your information in specific ways in order for the research to be reliable and accurate. If you withdraw from the study, we will keep the information about you that we have already obtained. To safeguard your rights, we will use the minimum personally-identifiable information possible. Under the provisions of the EU General Data Protection Regulation you have the right to know what information the CompARE Study Office has recorded about you. If you wish to find out more please contact the Data Protection Officer at the address below.

The Data Protection Officer
Legal Services
University of Birmingham
Edgbaston
Birmingham B15 2TT
Email: dataprotection@contacts.bham.ac.uk

## Will my GP be involved?

It is important that your GP is kept up to date with any treatment you are receiving. With your permission your GP will be informed that you are taking part in this study.

## What will happen to any samples I give?

Routine full blood test and other safety laboratory tests will be performed at your hospital laboratory throughout the course of the study to see how the medication is affecting your body.

When you had the biopsy to confirm your diagnosis, your tissue was analysed for Human Papillomavirus status by a specialist team to decide if you were suitable for the study. With your permission, we would like to collect tissue from your diagnostic biopsy and remaining samples from any surgery you may have whichever Group you are in. Your tissue samples will be identified by your Trial Number, initials and date of birth and will be sent for storage to the University of Birmingham for future research.

Blood and oral fluid samples collected as part of CompARE Collect will be also sent for storage to the University of Birmingham. Blood and oral fluid samples used will only be identified by your Trial Number, initials and date of birth.

Tissue, blood and oral fluid samples will be stored indefinitely for future research studies at the University of Birmingham in accordance with their local policy. Any future research studies will be approved by the Research Ethics Committee. Samples can be stored for use in future ethically and scientifically approved research in the UK or overseas, including genetic studies, which may use animals or in vitro models and research involving private or commercial companies. We cannot describe what these future projects might involve but we will look into the way cancer develops, respond to treatment and ways in which we may predict which tumour would respond best to which treatments. This can include genetic studies looking at mechanism of action of the tumour.

## What will happen to the results of the study?

We intend to publish the results of this research in a respected scientific or medical journal. No patients will be identified in any presentations, reports or publications resulting from the study. Patients taking part in this study can find out about the results from their study doctor once the results have been published if they have provided consent. A lay summary of the study results approved by the Research Ethics Committee will be available to you if you wish. The results will also be available on the CancerHelp website.

## Will any genetic tests be done?

We intend to study the expression of genes within the tissue, oral fluid and blood samples that you donate to help us identify why cancers develop and to find new treatments for them. In the future it may also be very important to use these samples for new research on tumour genes and related areas of research, such as the immune system.

## Who is organising and funding the research?

The study is sponsored by University of Birmingham and co-ordinated by the Cancer Research UK Clinical Trials Unit at the University of Birmingham.
Financial support is being provided by the Cancer Research UK, Clinical Trials Awards & Advisory Committee. A pharmaceutical company called AstraZeneca are suppling durvalumab for this study. Your study doctor or research nurse will not receive any payments for including you in this study.

## Who has reviewed the study?

All research in the NHS is looked at by an independent group of people, called a Research Ethics Committee, to protect your safety, rights, wellbeing and dignity. This study has been reviewed and given favourable opinion by the West Midlands- Solihull Research Ethics Committee and also by the Research and Development Office at your hospital.

## Further information and contact details

## What happens now?

You will have some time to think about the study and make your decision. You may wish to discuss it with your family or friends. If you take part, you will receive a copy of this information sheet and a copy of the signed consent form to take home. We will also inform your GP of your decision to take part in the study. If, at any time, you have any questions about the study you should contact your study doctor or research nurse using the details below.

## Local contact details:

Study Doctor: ____________________________________________

Research Nurse: ____________________________________________

☎: ____________________________________________

Emergency (24 hours)
☎: ____________________________________________

You may also find it helpful to contact the following organisations:

Your local Patient Advice and Liaison Service (PALS) or local equivalent who provide advice and support to patients, their families and their carers; website: http://www.nhs.uk
The contact number of your local Patient Advice and Liaison Service or local equivalent is:

______________________________________________________________________

For information about cancer and cancer research studies by Cancer Research UK contact CancerHelp. Information about CompARE can also be found on the CancerHelp website.

**Freephone: 0808 800 40 40** **Website: www.cancerhelp.org.uk**

**http://www.cancerresearchuk.org/about-cancer/find-a-clinical-trial**

To be printed on hospital headed paper

**Trial Summary Sheet**

**Phase III randomised controlled trial Comparing Alternative Regimens for escalating treatment of intermediate and high-risk oropharyngeal cancer**
EudraCT No.: 2014-003389-26
IRAS No.: 161147

We would like to invite you to take part in a non-commercial clinical trial (research study) run by the Cancer Research UK Clinical Trials Unit, University of Birmingham called CompARE. This is a short summary about the research study. You will be given a detailed Patient Information Sheet if tests show that you are suitable for the trial and if you are willing to participate.

## Oropharyngeal Cancer

Oropharyngeal cancer is a disease in which malignant cells form in the tissue of the oropharynx (throat). The oropharynx is the middle part of the throat including the base of the tongue, the tonsils, the soft palate, the uvula, and the walls of the throat. This type of cancer is usually treated with a combination of chemotherapy and radiotherapy (chemoradiotherapy), which is considered the standard treatment in the UK. The main causes of oropharyngeal cancer are: Tobacco smoking or drinking alcohol and the Human Papillomavirus, also known as HPV.

The cause of oropharyngeal cancer will affect how well patients respond to treatment. It is now recognised that there are two main types of oropharyngeal cancer:

**Low-risk oropharyngeal cancer:** about one third of oropharyngeal cancers are classified as low-risk. These are patients whose cancers are caused by the Human Papillomavirus and who do not smoke or smoke very little. These low-risk oropharyngeal cancers appear to respond very well to chemoradiotherapy which has a cure rate of more than 90%.

**Higher-risk (includes high-risk and intermediate-risk) oropharyngeal cancer:** the other two thirds of oropharyngeal cancer are classified as higher-risk. These are patients whose cancers are caused mainly by heavy tobacco smoking or alcohol intake. About half of these higher-risk cancers also have Human Papillomavirus in them. These higher-risk oropharyngeal cancers respond less well to the chemoradiotherapy, with cure rates of about 50-70%.

**Trial Summary Sheet**

## Purpose of the study

The CompARE study has been set up to investigate which treatment is most effective for patients who have higher-risk oropharyngeal cancer. The purpose of this study is to consider using an additional treatment in conjunction with standard chemoradiotherapy to increase cure rates of higher-risk oropharyngeal cancer. This will be done by adding immunotherapy to the standard chemoradiotherapy. Immunotherapy is novel approach, which uses drugs that encourages the body's natural defence system, the immune system to attack cancer cells.

## Why are you being asked?

You are being asked if you would like to take part in this study because you have been diagnosed with oropharyngeal cancer that we believe to be 'Higher risk'.

## Human Papillomavirus testing

If you decide to take part in the initial investigations, you will be asked to sign Informed Consent Form A. Once you have done this, your study doctor will register your intent to take part in the study with the CompARE Trial Office at the Cancer Research UK Clinical Trials Unit, University of Birmingham. You will then be assigned a unique Trial Number.

Sections of your diagnostic biopsy that showed you have oropharyngeal cancer will be sent to the Central Laboratory Services based at Newcastle Hospitals NHS Foundation Trust. Sections will be labelled with your Trial Number, initials and date of birth. Tests for Human Papillomavirus will be performed to check if the tumour is Human Papillomavirus related. If the tests show that you are eligible we will talk to you about taking part in the CompARE study and provide you with more information about what this involves.

## Study treatment

The CompARE study is investigating whether patients with higher-risk oropharyngeal cancer will benefit from additional treatment. If you decide to take part in the study, you should be willing to undergo either of two possible treatments shown in the table below. You will be randomly allocated to one of the two treatment options by the Cancer Research UK Clinical Trials Unit, University of Birmingham. To make sure there is no bias in the study, neither you nor your doctor will be able to choose which treatment you get.

**Treatment Options**

<table>
<tr><th colspan="2">Group 1: Chemoradiotherapy</th></tr>
<tr><td>Radiotherapy</td><td>Delivered 5 days a week for 7 weeks</td></tr>
<tr><td>Chemotherapy</td><td>Cisplatin will be given via a thin tube through a vein in your arm either:<br>• Once every three weeks for three cycles (given on days 1, 22 and 43 from start of radiotherapy) usually as a 10 hour infusion during inpatient clinic and may require an overnight stay.<br>OR<br>• Once a week for 7 weeks (given on days 1, 8, 15, 22, 29, 36 and 43 from start of radiotherapy) usually as a 3 hour infusion during outpatient visit.<br><br>Extra medication and hydration therapy may also be given before or after chemoradiotherapy to reduce the risk of sickness and other side effects as per standard practice.</td></tr>
<tr><td colspan="2">We will reassess the tumour 12 weeks after the end of the treatment and there may be the need to have an operation of the neck to remove any involved neck glands that did not respond to treatment.</td></tr>
</table>

| Group 5: Immunotherapy plus chemoradiotherapy | |
|---|---|
| **Induction Immunotherapy*** | First single dose of immunotherapy drug, durvalumab called induction durvalumab is delivered via 1 hour infusion. It is delivered via a thin tube through a vein in your arm. |
| **Radiotherapy** | As per Group 1, given after the end of the induction durvalumab. |
| **Chemotherapy** | As per Group 1, given after the end of the induction durvalumab. |
| **Adjuvant Immunotherapy**** | Durvalumab is delivered following chemoradiotherapy via 1 hour infusion. It is delivered via a thin tube through a vein in your arm and repeated every four weeks for 6 months. |
| We will reassess the tumour 12 weeks after the end of the chemoradiotherapy treatment and there may be the need to have an operation of the neck to remove any involved neck glands that did not respond to treatment. | |

*Definition of Induction: The first treatment given for a disease

**Definition of Adjuvant: Additional cancer treatment given after the primary treatment to enhance effectiveness of treatment or to lower the risk of cancer coming back

## Do I have to take part in these studies?

No. Taking part is entirely voluntary. If you do not want to take part, your care will NOT be affected in any way.

## Will my taking part in the trial be kept confidential?

All information collected about your participation in the study will be subject to the EU General Data Protection Regulation and to the Data Protection Act 2018 for health and social care and will be kept strictly confidential.

For information about cancer and cancer research studies by Cancer Research UK contact CancerHelp.
Information about CompARE can also be found on the CancerHelp website.

**Freephone: 0808 800 40 40** **Website: www.cancerhelp.org.uk**

http://www.cancerresearchuk.org/about-cancer/find-a-clinical-trial

**Supplementary Appendix 3: Exemplar CompARE informed consent forms**

To be printed on hospital headed paper

**Informed Consent Form D**
Randomisation (Arms 1 and 5)

**Phase III randomised controlled trial Comparing Alternative Regimens for escalating treatment of intermediate and high-risk oropharyngeal cancer (CompARE)**

EudraCT No.: 2014-003389-26
IRAS No.:161147

**Site:** ____________________ **Patient's TNO:** ☐☐☐

**Investigator:** ____________________

**Please initial each box**

1. I confirm that I have read and understand the Patient Information Sheet (version number …………. dated …………) for the above trial. I have had the opportunity to consider the information, ask questions and have had these answered satisfactorily. ☐

2. I understand that my participation is voluntary and that I am free to withdraw at any time without giving any reason, without my medical care or legal rights being affected. ☐

3. I give permission for my initials, date of birth, hospital number and NHS/CHI number to be given to the CompARE Trial Office when I am randomised into the trial as well as a copy of this consent form. ☐

4. I understand that relevant sections of my medical notes and data collected during the trial may be looked at by individuals from the CompARE Trial Office, the Institute of Head and Neck Studies and Education (InHANSE), AstraZeneca, regulatory authorities, the Sponsor and/or NHS bodies, where it is relevant to my taking part in this research. I give permission for these individuals to have access to my records. ☐

Please continue on next page

5. I give permission for a copy of my radiotherapy treatment plan and associated treatment planning images (CT scans/MRI scans) to be monitored and stored by the national trials Quality Assurance team. I understand that this data will be anonymised prior to storage and may be used for future research in patients treated with radiotherapy

6. I understand that the CompARE Trial Office, may access information held by Cancer Registries, and/or the NHS Data Linkage and Extract Service Services e.g. Health and Social Care Information Centre to keep in touch with me and to follow up on my health status.

7. I understand that I will be required to complete questionnaires regarding the impact of the treatment and my quality of life.

8. I agree to my GP being informed of my participation in this trial.

9. I agree to take part in the CompARE trial.

**Optional:** **Yes** **No**

**Please initial box**

10. I give permission for a lay summary of the CompARE Trial results to be provided to me at the end of the study

11. I give my consent for relevant documents and data relating to my involvement in the study identified above to be transferred and held in the University of Birmingham

| **Name of patient** | **Signature** | **Date** |
|---|---|---|
| **Name of person taking consent**<br>You must have signed the<br>Site Signature & Delegation Log | **Signature** | **Date** |

This trial is funded by:

CANCER RESEARCH UK

CRCTU-ICF-QCD-001, version 2.0a

Original to be kept in the Investigator Site File, 1 copy in hospital notes, 1 copy to the patient, 1 copy to the CompARE Trial Office

# Informed Consent Form

CompARE Collect: Blood, Oral Fluid and Tissue Sample Collection & Storage

## Phase III randomised controlled trial Comparing Alternative Regimens for escalating treatment of intermediate and high-risk oropharyngeal cancer (CompARE)

EudraCT No.: 2014-003389-26
IRAS No.: 161147

**Site:** ______________________________ **Patient's TNO:** ☐☐☐

**Investigator:** ______________________________

**Please initial each box**

| | |
|---|---|
| 1. I confirm that I have read and understand the Patient Information Sheet **(version number …………. dated …………………..)** for the above trial. I have had the opportunity to consider the information, ask questions and have had these answered satisfactorily. | ☐ |
| 2. I understand that any blood, oral fluid and tissue samples that I donate will be labelled with my trial number, patient initials and date of birth and will be sent to the University of Birmingham for storage for research associated with this trial. | ☐ |
| 3. I consent that research may include genetic and other current and future tests and analyses of my samples aimed at understanding the factors that influence my type of cancer and its treatment. I understand that I will not be informed of the results of these studies. | ☐ |
| 4. I give permission for a copy of this consent form to be given to the CompARE Trial Office when I am randomised into the trial. | ☐ |

***Please continue on next page***

5. I understand that my participation is voluntary and that I am free to withdraw at any time without giving any reason, without my medical care or legal rights being affected.

6. I agree to continue participating in the above trial.

**Optional:** **Yes** **No**

**Please initial box**

7. I agree to provide samples of blood for research associated with this trial. This is in addition to collection of routine blood samples. I understand that giving a blood sample is a gift for this research, is voluntary and that I am free to withdraw my approval for use of the sample at any time without giving a reason and without my medical treatment or legal rights being affected by this voluntary donation.

8. I agree to provide samples of oral fluid for research associated with this trial. I understand that giving an oral fluid sample is a gift for this research, is voluntary and that I am free to withdraw my approval for use of the sample at any time without giving a reason and without my medical treatment or legal rights being affected by this voluntary donation.

9. I agree to the collection of tissue from both my diagnostic biopsy and remaining samples from any surgery or future biopsy being used for research purposes. I understand that giving a tissue sample is a gift for this research, is voluntary and that I am free to withdraw my approval for use of the sample at any time without giving a reason and without my medical treatment or legal rights being affected by this voluntary donation.

10. I agree that my samples and the information gathered about me can be stored for possible use in future ethically and scientifically approved research in the UK or overseas, including genetic studies, which may use animals or in vitro models, and research involving private or commercial companies.

| **Name of patient** | **Signature** | **Date** |
|---|---|---|

| **Name of person taking consent** | **Signature** | **Date** |
|---|---|---|
| You must have signed the Site Signature & Delegation Log | | |

This trial is funded by:

CANCER RESEARCH UK

# Supplementary Appendix 4: Dose modification and toxicity management guidelines for immune-related, infusion-related, and non-immune-mediate reactions for durvalumab

## General Considerations regarding Immune-Mediated Reactions

| Dose Modifications | Toxicity Management |
|---|---|
| Drug administration modifications of study drug/study regimen will be made to manage potential immune-related AEs based on severity of treatment-emergent toxicities graded per NCI CTCAE v4.03 (unless indicated otherwise).<br>In addition to the criteria for permanent discontinuation of study drug/study regimen based on CTC grade/severity (table below), permanently discontinue study drug/study regimen for the following conditions:<br>• Inability to reduce corticosteroid to a dose of ≤10 mg of prednisone per day (or equivalent) within 12 weeks of the start of the immune-mediated adverse event (imAE)<br>• Grade 3 recurrence of a previously experienced treatment-related imAE following resumption of dosing<br>Grade 1 No dose modification<br>Grade 2 Hold study drug/study regimen dose until Grade 2 resolution to Grade ≤1.<br>If toxicity worsens, then treat as Grade 3 or Grade 4.<br>Study drug/study regimen can be resumed once | It is recommended that management of immune-mediated adverse events (imAEs) follows the guidelines presented in this table:<br>−It is possible that events with an inflammatory or immune mediated mechanism could occur in nearly all organs, some of them not noted specifically in these guidelines.<br>−Whether specific immune-mediated events (and/or laboratory indicators of such events) are noted in these guidelines or not, patients should be thoroughly evaluated to rule out any alternative aetiology (e.g., disease progression, concomitant medications, and infections) to a possible immune-mediated event. In the absence of a clear alternative aetiology, all such events should be managed as if they were immune related. General recommendations follow.<br>−Symptomatic and topical therapy should be considered for low grade (Grade 1 or 2, unless otherwise specified) events.<br>−For persistent (>3 to 5 days) low-grade (Grade 2) or severe (Grade ≥3) events, promptly start prednisone 1 to 2 mg/kg/day PO or IV equivalent. |

| Dose Modifications | Toxicity Management |
| --- | --- |
| event stabilizes to Grade ≤1 after completion of steroid taper.<br>Patients with endocrinopathies who may require prolonged or continued steroid replacement can be retreated with study drug/study regimen on the following conditions:<br>The event stabilizes and is controlled.<br>The patient is clinically stable as per Investigator or treating physician's clinical judgement.<br>Doses of prednisone are at ≤10 mg/day or equivalent.<br>Grade 3 Depending on the individual toxicity, study drug/study regimen may be permanently discontinued. Please refer to guidelines below.<br>Grade 4 Permanently discontinue study drug/study regimen.<br><br>Note: For asymptomatic amylase or lipase levels of >2.0×ULN, hold study drug/study regimen, and if complete work up shows no evidence of pancreatitis, study drug/study regimen may be continued or resumed.<br><br>Note: Study drug/study regimen should be permanently discontinued in Grade 3 events with high likelihood for morbidity and/or mortality – e.g., myocarditis, or other similar events even if they are not currently noted in the guidelines. Similarly, | −Some events with high likelihood for morbidity and/or mortality – e.g., myo-carditis, or other similar events even if they are not currently noted in the guidelines – should progress rapidly to high dose IV corticosteroids (methylprednisolone at 2 to 4 mg/kg/day) even if the event is Grade 2, and if clinical suspicion is high and/or there has been clinical confirmation. Consider, as necessary, discussing with the study physician, and promptly pursue specialist consultation.<br>−If symptoms recur or worsen during corticosteroid tapering (28 days of taper), increase the corticosteroid dose (prednisone dose [e.g., up to 2 to 4 mg/kg/day PO or IV equivalent]) until stabilization or improvement of symptoms, then resume corticosteroid tapering at a slower rate (>28 days of taper).<br>−More potent immunosuppressives such as TNF inhibitors (e.g., infliximab) (also refer to the individual sections of the imAEs for specific type of immunosuppressive) should be considered for events not responding to systemic steroids. Progression to use of more potent immunosuppressives should proceed more rapidly in events with high likelihood for morbidity and/or mortality – e.g., myocarditis, or other similar events even if they are not currently noted in the guidelines – when these events are not responding to systemic steroids. |

| Dose Modifications | Toxicity Management |
|---|---|
| consider whether study drug/study regimen should be permanently discontinued in Grade 2 events with high likelihood for morbidity and/or mortality – e.g., myocarditis, or other similar events even if they are not currently noted in the guidelines – when they do not rapidly improve to Grade <1 upon treatment with systemic steroids and following full taper<br>Note: There are some exceptions to permanent discontinuation of study drug for Grade 4 events (i.e., hyperthyroidism, hypothyroidism, type 1 diabetes mellitus). | −With long-term steroid and other immunosuppressive use, consider need for Pneumocystis jirovecii pneumonia (PJP, formerly known as Pneumocystis carinii pneumonia) prophylaxis, gastrointestinal protection, and glucose monitoring.<br>−Discontinuation of study drug/study regimen is not mandated for Grade 3/Grade 4 inflammatory reactions attributed to local tumor response (e.g., inflammatory reaction at sites of metastatic disease and lymph nodes). Continuation of study drug/study regimen in this situation should be based upon a benefit-risk analysis for that patient. |

AE, Adverse event; CTC, Common Toxicity Criteria; CTCAE, Common Terminology Criteria for Adverse Events; imAE, immune-mediated adverse event; IV, intravenous; NCI, National Cancer Institute; PO, By mouth.

## Specific Immune-Mediated Reactions

| Adverse Events | Severity Grade of the Event (NCI CTCAE version 4.0) | Dose Modifications | Toxicity Management |
|---|---|---|---|
| **Pneumonitis/ Interstitial Lung Disease (ILD)** | **Any Grade** | **General Guidance** | **For Any Grade:**<br>− Monitor patients for signs and symptoms of pneumonitis or ILD (new onset or worsening shortness of breath or cough). Patients should be evaluated with imaging and pulmonary function tests, including other diagnostic procedures as described below.<br>−Initial work-up may include clinical evaluation, monitoring of oxygenation via pulse oximetry (resting and exertion), laboratory work-up, and high-resolution CT scan. |
| | **Grade 1** (asymptomatic, clinical or diagnostic observations only; intervention not indicated) | No dose modifications required. However, consider holding study drug/study regimen dose as clinically appropriate and during diagnostic work-up for other etiologies. | **For Grade 1 (radiographic changes only):**<br>−Monitor and closely follow up in 2 to 4 days for clinical symptoms, pulse oximetry (resting and exertion), and laboratory work-up and then as clinically indicated.<br>−Consider Pulmonary and Infectious disease consult. |
| | **Grade 2** (symptomatic; medical intervention indicated; limiting instrumental ADL) | Hold study drug/study regimen dose until Grade 2 resolution to Grade ≤1.<br>• If toxicity worsens, then treat as Grade 3 or Grade 4.<br>•If toxicity improves to Grade ≤1, then the decision to reinitiate study drug/study regimen will be based upon treating physician's clinical judgment and after completion of steroid taper. | **For Grade 2 (mild to moderate new symptoms):**<br>−Monitor symptoms daily and consider hospitalization.<br>−Promptly start systemic steroids (e.g., prednisone 1 to 2 mg/kg/day PO or IV equivalent).<br>−Reimage as clinically indicated.<br>−If no improvement within 3 to 5 days, additional workup should be considered and prompt treatment with IV methylprednisolone 2 to 4 mg/kg/day started<br>−If still no improvement within 3 to 5 days despite IV methylprednisolone at 2 |

| Adverse Events | Severity Grade of the Event (NCI CTCAE version 4.0) | Dose Modifications | Toxicity Management |
|---|---|---|---|
| | | | to 4 mg/kg/day, promptly start immunosuppressive therapy such as TNF inhibitors (e.g., infliximab at 5 mg/kg every 2 weeks). Caution: It is important to rule out sepsis and refer to infliximab label for general guidance before using infliximab.<br>−Once the patient is improving, gradually taper steroids over ≥28 days and consider prophylactic antibiotics, antifungals, or anti-PJP treatment (refer to current NCCN guidelines for treatment of cancer-related infections)[a]<br>−Consider Pulmonary and Infectious Disease consult.<br>−Consider, as necessary, discussing with study physician. |
| | **Grade 3 or 4**<br>(Grade 3: severe symptoms; limiting self-care ADL; oxygen indicated)<br>(Grade 4: life-threatening respiratory compromise; urgent intervention indicated [e.g., tracheostomy or intubation]) | Permanently discontinue study drug/study regimen. | **For Grade 3 or 4 (severe or new symptoms, new/worsening hypoxia, life-threatening):**<br>−Promptly initiate empiric IV methylprednisolone 1 to 4 mg/kg/day or equivalent.<br>−Obtain Pulmonary and Infectious Disease consults; consider, as necessary, discussing with study physician.<br>−Hospitalize the patient.<br>−Supportive care (e.g., oxygen).<br>−If no improvement within 3 to 5 days, additional workup should be considered and prompt treatment with additional immunosuppressive therapy such as TNF inhibitors (e.g., infliximab at 5 mg/kg every 2 weeks' dose) started. Caution: rule out sepsis and refer to infliximab label for general guidance before using infliximab.<br>−Once the patient is improving, |

| Adverse Events | Severity Grade of the Event (NCI CTCAE version 4.0) | Dose Modifications | Toxicity Management |
|---|---|---|---|
| | | | gradually taper steroids over ≥28 days and consider prophylactic antibiotics, antifungals, and, in particular, anti-PJP treatment (refer to current NCCN guidelines for treatment of cancer-related infections).[a] |
| **Diarrhoea/ Colitis**<br><br>**Large intestine perforation/Intestine perforation** | **Any Grade** | **General Guidance** | **For Any Grade:**<br>−Monitor for symptoms that may be related to diarrhoea/enterocolitis (abdominal pain, cramping, or changes in bowel habits such as increased frequency over baseline or blood in stool) or related to bowel perforation (such as sepsis, peritoneal signs, and ileus).<br>−When symptoms or evaluation indicate a perforation is suspected, consult a surgeon experienced in abdominal surgery immediately without any delay<br>−Patients should be thoroughly evaluated to rule out any alternative aetiology (e.g., disease progression, other medications, or infections), including testing for clostridium difficile toxin, etc.<br>−Steroids should be considered in the absence of clear alternative aetiology, even for low-grade events, in order to prevent potential progression to higher grade event, including perforation.<br>−Use analgesics carefully; they can mask symptoms of perforation and peritonitis. |

| Adverse Events | Severity Grade of the Event (NCI CTCAE version 4.0) | Dose Modifications | Toxicity Management |
|---|---|---|---|
| | **Grade 1**<br><br>(Diarrhoea: stool frequency of <4 over baseline per day)<br><br>(Colitis: asymptomatic; clinical or diagnostic observations only) | No dose modifications. | **For Grade 1:**<br><br>−Monitor closely for worsening symptoms.<br><br>−Consider symptomatic treatment, including hydration, electrolyte replacement, dietary changes (e.g., American Dietetic Association colitis diet), and loperamide. Use probiotics as per treating physician's clinical judgment. |
| | **Grade 2**<br><br>(Diarrhoea: stool frequency of 4 to 6 over baseline per day) (Colitis: abdominal pain; mucus or blood in stool)<br><br>(Perforation: symptomatic; medical intervention indicated*)<br><br>*"medical intervention" is not invasive | Hold study drug/study regimen until resolution to Grade ≤1<br><br>−If toxicity worsens, then treat as Grade 3 or Grade 4.<br><br>−If toxicity improves to Grade ≤1, then study drug/study regimen can be resumed after completion of steroid taper. | **For Grade 2:**<br><br>−Consider symptomatic treatment, including hydration, electrolyte replacement, dietary changes (e.g., American Dietetic Association colitis diet), and loperamide and/or budesonide.<br><br>−Promptly start prednisone 1 to 2 mg/kg/day PO or IV equivalent.<br><br>−If event is not responsive within 3 to 5 days or worsens despite prednisone at 1 to 2 mg/kg/day PO or IV equivalent, GI consult should be obtained for consideration of further workup, such as imaging and/or colonoscopy, to confirm colitis and rule out perforation, and prompt treatment with IV methylprednisolone 2 to 4 mg/kg/day started.<br><br>−If still no improvement within 3 to 5 days despite 2 to 4 mg/kg IV methylprednisolone, promptly start immunosuppressives such as infliximab at 5 mg/kg once every 2 weeks. Caution: it is important to rule out bowel perforation and refer to infliximab label for general guidance before using infliximab.<br><br>−Consider, as necessary, discussing |

| Adverse Events | Severity Grade of the Event (NCI CTCAE version 4.0) | Dose Modifications | Toxicity Management |
|---|---|---|---|
| | | | with study physician if no resolution to Grade ≤1 in 3 to 4 days.<br>−Once the patient is improving, gradually taper steroids over ≥28 days and consider prophylactic antibiotics, antifungals, and anti-PJP treatment (refer to current NCCN guidelines for treatment of cancer-related infections).[a] |
| | **Grade 3 or 4**<br>(Grade 3 Diarrhea: stool frequency of ≥7 over baseline per day;<br>Grade 4 Diarrhea: life threatening consequences)<br>(Grade 3 Colitis: severe abdominal pain, change in bowel habits, medical intervention indicated, peritoneal signs;<br>Grade 4 Colitis: life-threatening consequences, urgent intervention indicated)<br>(Grade 3 Perforation: severe symptoms, elective* operative intervention indicated; Grade | **Grade 3**<br>Permanently discontinue study drug/study regimen for Grade 3 if toxicity does not improve to Grade ≤1 within 14 days; study drug/study regimen can be resumed after completion of steroid taper.<br>**Grade 4**<br>Permanently discontinue study drug/study regimen. | **For Grade 3 or 4:**<br>−Promptly initiate empiric IV methylprednisolone 2 to 4 mg/kg/day or equivalent.<br>−Monitor stool frequency and volume and maintain hydration.<br>−Urgent GI consult and imaging and/or colonoscopy as appropriate.<br>−If still no improvement within 3 to 5 days of IV methylprednisolone 2 to 4 mg/kg/day or equivalent, promptly start further immunosuppressives (e.g., infliximab at 5 mg/kg once every 2 weeks). **Caution**: Ensure GI consult to rule out bowel perforation and refer to infliximab label for general guidance before using infliximab. If perforation is suspected, consult a surgeon experienced in abdominal surgery immediately without any delay.<br>−Once the patient is improving, gradually taper steroids over ≥28 days and consider prophylactic antibiotics, antifungals, and anti-PJP treatment (refer to current NCCN guidelines for treatment of cancer-related infections ).[a] |

| Adverse Events | Severity Grade of the Event (NCI CTCAE version 4.0) | Dose Modifications | Toxicity Management |
|---|---|---|---|
| | 4 Perforation: life-threatening consequences, urgent intervention indicated)<br><br>*This guidance anticipates that Grade 3 operative interventions of perforations are usually not elective | | |
| **Hepatitis**<br><br>(elevated LFTs)<br><br>Infliximab should not be used for management of immune-related hepatitis. | **Any Elevations in AST, ALT or TB as Described Below** | **General Guidance** | **For Any Elevations Described:**<br><br>−Monitor and evaluate liver function test: AST, ALT, ALP, and TB.<br><br>Evaluate for alternative etiologies (e.g., viral hepatitis, disease progression, concomitant medications). |
| | | −No dose modifications.<br><br>−If it worsens, then treat as described for elevations in the row below | |
| | **AST or ALT >3.0×ULN and ≤5.0×ULN if baseline normal, >3-5×baseline if baseline abnormal; and/or TB >1.5×ULN and ≤3.0×ULN if baseline** | −Hold study drug/study regimen dose until resolution to AST or ALT ≤3.0×ULN and/or TB ≤1.5×ULN if baseline normal, or to AST or ALT ≤3.0×baseline and/or TB ≤1.5×baseline if baseline abnormal. | −If no resolution to AST or ALT ≤3.0×ULN and/or TB ≤1.5×ULN if baseline normal, or to AST or ALT ≤3.0×baseline and/or TB ≤1.5×baseline if baseline abnormal, in 1 to 2 days, consider, as necessary, discussing with study physician.<br><br>−If event is persistent (>3 to 5 days) or worsens, promptly start prednisone 1 to |

| Adverse Events | Severity Grade of the Event (NCI CTCAE version 4.0) | Dose Modifications | Toxicity Management |
|---|---|---|---|
| | **normal, >1.5-3.0×baseline if baseline abnormal** | −If toxicity worsens, then treat as described for elevation in the row below.<br>−If toxicity improves to AST or ALT ≤3.0×ULN and/or TB ≤1.5×ULN if baseline normal, or to AST or ALT ≤3.0×baseline and/or TB ≤1.5×baseline if baseline abnormal, resume study drug/study regimen after completion of steroid taper. | 2 mg/kg/day PO or IV equivalent.<br>−If still no improvement within 3 to 5 days despite 1 to 2 mg/kg/day of prednisone PO or IV equivalent, consider additional work up and start prompt treatment with IV methylprednisolone 2 to 4 mg/kg/day.<br>−If still no improvement within 3 to 5 days despite 2 to 4 mg/kg/day of IV methylprednisolone, promptly start immunosuppressives (i.e., mycophenolate mofetil).[a] Discuss with study physician if mycophenolate mofetil is not available. **Infliximab should NOT be used.**<br>−Once the patient is improving, gradually taper steroids over ≥28 days and consider prophylactic antibiotics, antifungals, and anti-PJP treatment (refer to current NCCN guidelines for treatment of cancer-related infections).[a] |
| | **AST or ALT >5.0×ULN if baseline normal, >5×baseline if baseline abnormal; and/or TB >3.0×ULN if baseline normal; >3.0×baseline if baseline abnormal** | −Hold study drug/study regimen dose until resolution to AST or ALT ≤3.0×ULN and/or TB ≤1.5×ULN if baseline normal, or to AST or ALT ≤3.0×baseline and/or TB ≤1.5×baseline if baseline abnormal<br>−Resume study drug/study regimen if elevations downgrade to AST or ALT ≤3.0×ULN and/or TB ≤1.5×ULN if | −If still no improvement within 3 to 5 days despite 1 to 4 mg/kg/day methylprednisolone IV or equivalent, promptly start treatment with immunosuppressive therapy (i.e., mycophenolate mofetil). Discuss with study physician if mycophenolate is not available. **Infliximab should NOT be used.**<br>−Request Hepatology consult, and perform abdominal workup and imaging as appropriate.<br>−Once the patient is improving, gradually taper steroids over ≥28 days |

| Adverse Events | Severity Grade of the Event (NCI CTCAE version 4.0) | Dose Modifications | Toxicity Management |
|---|---|---|---|
| | | baseline normal, or to AST or ALT ≤3.0×baseline and/or TB ≤1.5×baseline if baseline abnormal, within 14 days and after completion of steroid taper.<br><br>−Permanently discontinue study drug/study regimen if the elevations do not downgrade as described in bullet above within 14 days<br><br>−For elevations in transaminases >8×ULN or elevations in TB >5×ULN if baseline normal, or for elevations in transaminases >8×baseline and/or TB >5×baseline if baseline abnormal, permanently discontinue study drug/study regimen.<br><br>Permanently discontinue study drug/study regimen for any case meeting Hy's law criteria (AST and/or ALT >3 × ULN + bilirubin >2 × ULN without initial findings of cholestasis (i.e., elevated alkaline P04) and in the absence of any alternative cause.[b] | and consider prophylactic antibiotics, antifungals, and anti-PJP treatment (refer to current NCCN guidelines for treatment of cancer-related infections).[a] |

| Adverse Events | Severity Grade of the Event (NCI CTCAE version 4.0) | Dose Modifications | Toxicity Management |
|---|---|---|---|
| **Nephritis or renal dysfunction**<br>(elevated serum creatinine) | **Any Grade** | **General Guidance** | **For Any Grade**<br>−Consult with nephrologist.<br>−Monitor for signs and symptoms that may be related to changes in renal function (e.g., routine urinalysis, elevated serum BUN and creatinine, decreased creatinine clearance, electrolyte imbalance, decrease in urine output, or proteinuria).<br>−Patients should be thoroughly evaluated to rule out any alternative aetiology (e.g., disease progression or infections).<br>−Steroids should be considered in the absence of clear alternative aetiology even for low-grade events (Grade 2), in order to prevent potential progression to higher grade event. |
| | **Grade 1**<br>(Serum creatinine > 1 to 1.5 × baseline; > ULN to 1.5 × ULN) | No dose modifications. | **For Grade 1:**<br>−Monitor serum creatinine weekly and any accompanying symptoms.<br>•If creatinine returns to baseline, resume its regular monitoring per study protocol.<br>•If creatinine worsens, depending on the severity, treat as Grade 2, 3, or 4.<br>−Consider symptomatic treatment, including hydration, electrolyte replacement, and diuretics. |
| | **Grade 2**<br>(serum creatinine >1.5 to 3.0 × baseline; >1.5 to 3.0 × ULN) | Hold study drug/study regimen until resolution to Grade ≤1 or baseline.<br>•If toxicity worsens, then treat as Grade 3 or 4.<br>•If toxicity improves to Grade ≤1 or baseline, then resume study drug/study regimen after | **For Grade 2:**<br>−Consider symptomatic treatment, including hydration, electrolyte replacement, and diuretics.<br>−Carefully monitor serum creatinine every 2 to 3 days and as clinically warranted.<br>−Consult nephrologist and consider |

| Adverse Events | Severity Grade of the Event (NCI CTCAE version 4.0) | Dose Modifications | Toxicity Management |
|---|---|---|---|
| | | completion of steroid taper. | renal biopsy if clinically indicated.<br>−If event is persistent (>3 to 5 days) or worsens, promptly start prednisone 1 to 2 mg/kg/day PO or IV equivalent.<br>−If event is not responsive within 3 to 5 days or worsens despite prednisone at 1 to 2 mg/kg/day PO or IV equivalent, additional workup should be considered and prompt treatment with IV methylprednisolone at 2 to 4 mg/kg/day started.<br>−Once the patient is improving, gradually taper steroids over ≥28 days and consider prophylactic antibiotics, antifungals, and anti-PJP treatment (refer to current NCCN guidelines for treatment of cancer-related infections).[a]<br>−When event returns to baseline, resume study drug/study regimen and routine serum creatinine monitoring per study protocol. |
| | **Grade 3 or 4**<br>(Grade 3: serum creatinine >3.0 × baseline; >3.0 to 6.0 × ULN;<br>Grade 4: serum creatinine >6.0 × ULN) | Permanently discontinue study drug/study regimen. | **For Grade 3 or 4:**<br>−Carefully monitor serum creatinine on daily basis.<br>−Consult nephrologist and consider renal biopsy if clinically indicated.<br>−Promptly start prednisone 1 to 2 mg/kg/day PO or IV equivalent.<br>−If event is not responsive within 3 to 5 days or worsens despite prednisone at 1 to 2 mg/kg/day PO or IV equivalent, additional workup should be considered and prompt treatment with IV methylprednisolone 2 to 4 mg/kg/day started.<br>−Once the patient is improving, gradually taper steroids over ≥28 days and consider prophylactic antibiotics, antifungals, and anti-PJP treatment |

| Adverse Events | Severity Grade of the Event (NCI CTCAE version 4.0) | Dose Modifications | Toxicity Management |
|---|---|---|---|
| | | | (refer to current NCCN guidelines for treatment of cancer-related infections).[a] |
| **Rash or Dermatitis** (including Pemphigoid) | **Any Grade** (refer to NCI CTCAE v 4.03 for definition of severity/grade depending on type of skin rash) | **General Guidance** | **For Any Grade**<br>−Monitor for signs and symptoms of dermatitis (rash and pruritus).<br>−IF THERE IS ANY BULLOUS FORMATION, THE STUDY PHYSICIAN SHOULD BE CONTACTED AND STUDY DRUG DISCONTINUED IF SUSPECT STEVENS-JOHNSON SYNDROME OR TOXIC EPIDERMAL NECROLYSIS. |
| | **Grade 1** | No dose modifications | **For Grade 1:**<br>−Consider symptomatic treatment, including oral antipruritics (e.g., diphenhydramine or hydroxyzine) and topical therapy (e.g., urea cream). |
| | **Grade 2** | For persistent (>1 to 2 weeks) Grade 2 events, hold scheduled study drug/study regimen until resolution to Grade ≤1 or baseline.<br>•If toxicity worsens, then treat as Grade 3.<br>•If toxicity improves to Grade ≤1 or baseline, then resume drug/study regimen after completion of steroid taper. | **For Grade 2:**<br>−Obtain Dermatology consult.<br>−Consider symptomatic treatment, including oral antipruritics (e.g., diphenhydramine or hydroxyzine) and topical therapy (e.g., urea cream).<br>−Consider moderate-strength topical steroid.<br>−If no improvement of rash/skin lesions occurs within 3 to 5 days or is worsening despite symptomatic treatment and/or use of moderate strength topical steroid, consider, as necessary, discussing with study physician and promptly start systemic steroids such as prednisone 1 to 2 mg/kg/day PO or IV equivalent.<br>−Consider skin biopsy if the event is persistent for >1 to 2 weeks or recurs. |

| Adverse Events | Severity Grade of the Event (NCI CTCAE version 4.0) | Dose Modifications | Toxicity Management |
|---|---|---|---|
| | **Grade 3 or 4** | **For Grade 3:**<br>Hold study drug/study regimen until resolution to Grade ≤1 or baseline.<br>If temporarily holding the study drug/study regimen does not provide improvement of the Grade 3 skin rash to Grade ≤1 or baseline within 30 days, then permanently discontinue study drug/study regimen.<br>**For Grade 4:**<br>Permanently discontinue study drug/study regimen. | **For Grade 3 or 4:**<br>−Consult Dermatology.<br>−Promptly initiate empiric IV methylprednisolone 1 to 4 mg/kg/day or equivalent.<br>−Consider hospitalization.<br>−Monitor extent of rash [Rule of Nines].<br>−Consider skin biopsy (preferably more than 1) as clinically feasible.<br>−Once the patient is improving, gradually taper steroids over ≥28 days and consider prophylactic antibiotics, antifungals, and anti-PJP treatment (refer to current NCCN guidelines for treatment of cancer-related infections).[a]<br>−Consider, as necessary, discussing with study physician. |
| **Endocrinopathy**<br>(e.g., hyperthyroidism, thyroiditis, hypothyroidism, Type 1 diabetes mellitus, hypophysitis, hypopituitarism, and adrenal insufficiency; exocrine event of amylase/lipase increased also included in this section) | **Any Grade**<br>(depending on the type of endocrinopathy, refer to NCI CTCAE v4.03 for defining the CTC grade/severity) | **General Guidance** | **For Any Grade:**<br>−Consider consulting an endocrinologist for endocrine events.<br>−Consider, as necessary, discussing with study physician.<br>−Monitor patients for signs and symptoms of endocrinopathies. Non-specific symptoms include headache, fatigue, behavior changes, changed mental status, vertigo, abdominal pain, unusual bowel habits, polydipsia, polyuria, hypotension, and weakness.<br>−Patients should be thoroughly evaluated to rule out any alternative aetiology (e.g., disease progression including brain metastases, or infections).<br>−Depending on the suspected endocrinopathy, monitor and evaluate thyroid function tests: TSH, free T3 and free T4 and other relevant endocrine |

| Adverse Events | Severity Grade of the Event (NCI CTCAE version 4.0) | Dose Modifications | Toxicity Management |
|---|---|---|---|
| | | | and related labs (e.g., blood glucose and ketone levels, HgA1c).<br>−For asymptomatic elevations in serum amylase and lipase >ULN and <3x ULN, corticosteroid treatment is not indicated as long as there are no other signs or symptoms of pancreatic inflammation.<br>−If a patient experiences an AE that is thought to be possibly of autoimmune nature (e.g., thyroiditis, pancreatitis, hypophysitis, or diabetes insipidus), the investigator should send a blood sample for appropriate autoimmune antibody testing. |
| | **Grade 1** | No dose modifications. | **For Grade 1 (including those with asymptomatic TSH elevation):**<br>−Monitor patient with appropriate endocrine function tests.<br>−For suspected hypophysitis/hypopituitarism, consider consultation of an endocrinologist to guide assessment of early-morning ACTH, cortisol, TSH and free T4; also consider gonadotropins, sex hormones, and prolactin levels, as well as cosyntropin stimulation test (though it may not be useful in diagnosing early secondary adrenal insufficiency).<br>−If TSH < 0.5 × LLN, or TSH >2 × ULN, or consistently out of range in 2 subsequent measurements, include free T4 at subsequent cycles as clinically indicated and consider consultation of an endocrinologist. |

| Adverse Events | Severity Grade of the Event (NCI CTCAE version 4.0) | Dose Modifications | Toxicity Management |
|---|---|---|---|
| | **Grade 2** | For Grade 2 endocrinopathy other than hypothyroidism and Type 1 diabetes mellitus, hold study drug/study regimen dose until patient is clinically stable.<br>−If toxicity worsens, then treat as Grade 3 or Grade 4.<br>Study drug/study regimen can be resumed once event stabilizes and after completion of steroid taper.<br>Patients with endocrinopathies who may require prolonged or continued steroid replacement (e.g., adrenal insufficiency) can be retreated with study drug/study regimen on the following conditions:<br>1. The event stabilizes and is controlled.<br>2. The patient is clinically stable as per investigator or treating physician's clinical judgement.<br>3. Doses of prednisone are ≤10 mg/day or equivalent. | **For Grade 2 (including those with symptomatic endocrinopathy):**<br>−Consult endocrinologist to guide evaluation of endocrine function and, as indicated by suspected endocrinopathy and as clinically indicated, consider pituitary scan.<br>−For all patients with abnormal endocrine work up, except those with isolated hypothyroidism or Type 1 DM, and as guided by an endocrinologist, consider short term corticosteroids (e.g., 1 to 2 mg/kg/day methylprednisolone or IV equivalent) and prompt initiation of treatment with relevant hormone replacement (e.g., hydrocortisone, sex hormones).<br>−Isolated hypothyroidism may be treated with replacement therapy, without study drug/study regimen interruption, and without corticosteroids.<br>−Isolated Type 1 diabetes mellitus (DM) may be treated with appropriate diabetic therapy, without study drug/study regimen interruption, and without corticosteroids.<br>−Once patients on steroids are improving, gradually taper immunosuppressive steroids (as appropriate and with guidance of endocrinologist) over ≥28 days and consider prophylactic antibiotics, antifungals, and anti-PJP treatment (refer to current NCCN guidelines for treatment of cancer-related infections).[a]<br>−For patients with normal endocrine workup (laboratory assessment or MRI scans), repeat laboratory assessments/MRI as clinically indicated. |

| Adverse Events | Severity Grade of the Event (NCI CTCAE version 4.0) | Dose Modifications | Toxicity Management |
|---|---|---|---|
| | **Grade 3 or 4** | For Grade 3 or 4 endocrinopathy other than hypothyroidism and Type 1 diabetes mellitus, hold study drug/study regimen dose until endocrinopathy symptom(s) are controlled.<br>Study drug/study regimen can be resumed once event stabilizes and after completion of steroid taper.<br>Patients with endocrinopathies who may require prolonged or continued steroid replacement (e.g., adrenal insufficiency) can be retreated with study drug/study regimen on the following conditions:<br>1. The event stabilizes and is controlled.<br>2. The patient is clinically stable as per investigator or treating physician's clinical judgement.<br>3. Doses of prednisone are ≤10 mg/day or equivalent. | **For Grade 3 or 4:**<br>−Consult endocrinologist to guide evaluation of endocrine function and, as indicated by suspected endocrinopathy and as clinically indicated, consider pituitary scan. Hospitalization recommended.<br>−For all patients with abnormal endocrine work up, except those with isolated hypothyroidism or Type 1 DM, and as guided by an endocrinologist, promptly initiate empiric IV methylprednisolone 1 to 2 mg/kg/day or equivalent, as well as relevant hormone replacement (e.g., hydrocortisone, sex hormones).<br>−For adrenal crisis, severe dehydration, hypotension, or shock, immediately initiate IV corticosteroids with mineralocorticoid activity.<br>−Isolated hypothyroidism may be treated with replacement therapy, without study drug/study regimen interruption, and without corticosteroids.<br>−Isolated Type 1 diabetes mellitus may be treated with appropriate diabetic therapy, without study drug/study regimen interruption, and without corticosteroids.<br>−Once patients on steroids are improving, gradually taper immunosuppressive steroids (as appropriate and with guidance of endocrinologist) over ≥28 days and consider prophylactic antibiotics, antifungals, and anti-PJP treatment (refer to current NCCN guidelines for treatment of cancer-related infections ).[a] |

| Adverse Events | Severity Grade of the Event (NCI CTCAE version 4.0) | Dose Modifications | Toxicity Management |
|---|---|---|---|
| **Neurotoxicity**<br><br>(to include but not be limited to limbic encephalitis and autonomic neuropathy, excluding Myasthenia Gravis and Guillain-Barre) | **Any Grade**<br><br>(depending on the type of neurotoxicity, refer to NCI CTCAE v4.03 for defining the CTC grade/severity) | **General Guidance** | **For Any Grade:**<br><br>−Patients should be evaluated to rule out any alternative aetiology (e.g., disease progression, infections, metabolic syndromes, or medications).<br><br>−Monitor patient for general symptoms (headache, nausea, vertigo, behavior change, or weakness).<br><br>−Consider appropriate diagnostic testing (e.g., electromyogram and nerve conduction investigations).<br><br>−Perform symptomatic treatment with Neurology consult as appropriate. |
| | **Grade 1** | No dose modifications. | **For Grade 1:**<br><br>−See "Any Grade" recommendations above. |
| | **Grade 2** | For acute motor neuropathies or neurotoxicity, hold study drug/study regimen dose until resolution to Grade ≤1.<br><br>For sensory neuropathy/neuropathic pain, consider holding study drug/study regimen dose until resolution to Grade ≤1.<br><br>If toxicity worsens, then treat as Grade 3 or 4.<br><br>Study drug/study regimen can be resumed once event improves to Grade ≤1 and after completion of steroid taper. | **For Grade 2:**<br><br>−Consider, as necessary, discussing with the study physician.<br><br>−Obtain Neurology consult.<br><br>−Sensory neuropathy/neuropathic pain may be managed by appropriate medications (e.g., gabapentin or duloxetine).<br><br>−Promptly start systemic steroids prednisone 1 to 2 mg/kg/day PO or IV equivalent.<br><br>−If no improvement within 3 to 5 days despite 1 to 2 mg/kg/day prednisone PO or IV equivalent, consider additional workup and promptly treat with additional immunosuppressive therapy (e.g., IV IG). |
| | **Grade 3 or 4** | **For Grade 3:**<br><br>Hold study drug/study regimen dose until | **For Grade 3 or 4:**<br><br>−Consider, as necessary, discussing with study physician. |

| Adverse Events | Severity Grade of the Event (NCI CTCAE version 4.0) | Dose Modifications | Toxicity Management |
| --- | --- | --- | --- |
| | | resolution to Grade ≤1.<br><br>Permanently discontinue study drug/study regimen if Grade 3 imAE does not resolve to Grade ≤1 within 30 days.<br><br>**For Grade 4:**<br><br>Permanently discontinue study drug/study regimen. | −Obtain Neurology consult.<br><br>−Consider hospitalization.<br><br>−Promptly initiate empiric IV methylprednisolone 1 to 2 mg/kg/day or equivalent.<br><br>−If no improvement within 3 to 5 days despite IV corticosteroids, consider additional workup and promptly treat with additional immunosuppressants (e.g., IV IG).<br><br>−Once stable, gradually taper steroids over ≥28 days. |
| **Peripheral neuromotor syndromes**<br><br>(such as Guillain-Barre and myasthenia gravis) | **Any Grade** | **General Guidance** | **For Any Grade:**<br><br>−The prompt diagnosis of immune-mediated peripheral neuromotor syndromes is important, since certain patients may unpredictably experience acute decompensations that can result in substantial morbidity or in the worst case, death. Special care should be taken for certain sentinel symptoms that may predict a more severe outcome, such as prominent dysphagia, rapidly progressive weakness, and signs of respiratory insufficiency or autonomic instability.<br><br>−Patients should be evaluated to rule out any alternative aetiology (e.g., disease progression, infections, metabolic syndromes or medications). It should be noted that the diagnosis of immune-mediated peripheral neuromotor syndromes can be particularly challenging in patients with underlying cancer, due to the multiple potential confounding effects of cancer (and its treatments) throughout the |

| Adverse Events | Severity Grade of the Event (NCI CTCAE version 4.0) | Dose Modifications | Toxicity Management |
|---|---|---|---|
| | | | neuraxis. Given the importance of prompt and accurate diagnosis, it is essential to have a low threshold to obtain a Neurology consult.<br>−Neurophysiologic diagnostic testing (e.g., electromyogram and nerve conduction investigations, and "repetitive stimulation" if myasthenia is suspected) are routinely indicated upon suspicion of such conditions and may be best facilitated by means of a Neurology consultation.<br>−It is important to consider that the use of steroids as the primary treatment of Guillain-Barre is not typically considered effective. Patients requiring treatment should be started with IV IG and followed by plasmapheresis if not responsive to IV IG. |
| | **Grade 1** | No dose modifications | **For Grade 1:**<br>−Consider, as necessary, discussing with the study physician.<br>−Care should be taken to monitor patients for sentinel symptoms of a potential decompensation as described above.<br>−Obtain a Neurology consult. |
| | **Grade 2** | Hold study drug/study regimen dose until resolution to Grade ≤1.<br>Permanently discontinue study drug/study regimen if it does not resolve to Grade ≤1 within 30 days or if there are signs of respiratory insufficiency or autonomic instability. | **For Grade 2:**<br>−Consider, as necessary, discussing with the study physician.<br>−Care should be taken to monitor patients for sentinel symptoms of a potential decompensation as described above.<br>−Obtain a Neurology consult<br>−Sensory neuropathy/neuropathic pain may be managed by appropriate |

| Adverse Events | Severity Grade of the Event (NCI CTCAE version 4.0) | Dose Modifications | Toxicity Management |
|---|---|---|---|
| | | | medications (e.g., gabapentin or duloxetine).<br>*MYASTHENIA GRAVIS:*<br>o Steroids may be successfully used to treat myasthenia gravis. It is important to consider that steroid therapy (especially with high doses) may result in transient worsening of myasthenia and should typically be administered in a monitored setting under supervision of a consulting neurologist.<br>o Patients unable to tolerate steroids may be candidates for treatment with plasmapheresis or IV IG. Such decisions are best made in consultation with a neurologist, taking into account the unique needs of each patient.<br>o If myasthenia gravis-like neurotoxicity is present, consider starting AChE inhibitor therapy in addition to steroids. Such therapy, if successful, can also serve to reinforce the diagnosis.<br>*GUILLAIN-BARRE:*<br>**o** It is important to consider here that the use of steroids as the primary treatment of Guillain-Barre is not typically considered effective.<br>o Patients requiring treatment should be started with IV IG and followed by plasmapheresis if not responsive to IV IG. |
| | **Grade 3 or 4** | **For Grade 3:**<br>Hold study drug/study regimen dose until resolution to Grade ≤1.<br>Permanently discontinue study drug/study regimen if Grade 3 imAE does not resolve to Grade ≤1 within | **For Grade 3 or 4 (severe or life-threatening events):**<br>−Consider, as necessary, discussing with study physician.<br>−Recommend hospitalization.<br>−Monitor symptoms and obtain neurological consult. |

| Adverse Events | Severity Grade of the Event (NCI CTCAE version 4.0) | Dose Modifications | Toxicity Management |
|---|---|---|---|
| | | 30 days or if there are signs of respiratory insufficiency or autonomic instability.<br>**For Grade 4:**<br>Permanently discontinue study drug/study regimen. | *MYASTHENIA GRAVIS:*<br>o Steroids may be successfully used to treat myasthenia gravis. They should typically be administered in a monitored setting under supervision of a consulting neurologist.<br>o Patients unable to tolerate steroids may be candidates for treatment with plasmapheresis or IV IG.<br>o If myasthenia gravis-like neurotoxicity present, consider starting AChE inhibitor therapy in addition to steroids. Such therapy, if successful, can also serve to reinforce the diagnosis.<br>*GUILLAIN-BARRE:*<br>o It is important to consider here that the use of steroids as the primary treatment of Guillain-Barre is not typically considered effective.<br>o Patients requiring treatment should be started with IV IG and followed by plasmapheresis if not responsive to IV IG. |
| **Myocarditis** | **Any Grade** | **General Guidance**<br>Discontinue drug permanently if biopsy-proven immune-mediated myocarditis. | **For Any Grade:**<br>−The prompt diagnosis of immune-mediated myocarditis is important, particularly in patients with baseline cardiopulmonary disease and reduced cardiac function.<br>−Consider, as necessary, discussing with the study physician.<br>−Monitor patients for signs and symptoms of myocarditis (new onset or worsening chest pain, arrhythmia, shortness of breath, peripheral edema). As some symptoms can overlap with lung toxicities, simultaneously evaluate for and rule out pulmonary toxicity as well as other causes (e.g., pulmonary |

| Adverse Events | Severity Grade of the Event (NCI CTCAE version 4.0) | Dose Modifications | Toxicity Management |
|---|---|---|---|
| | | | embolism, congestive heart failure, malignant pericardial effusion). A Cardiology consultation should be obtained early, with prompt assessment of whether and when to complete a cardiac biopsy, including any other diagnostic procedures.<br><br>−Initial work-up should include clinical evaluation, BNP, cardiac enzymes, ECG, echocardiogram (ECHO), monitoring of oxygenation via pulse oximetry (resting and exertion), and additional laboratory work-up as indicated. Spiral CT or cardiac MRI can complement ECHO to assess wall motion abnormalities when needed.<br><br>−Patients should be thoroughly evaluated to rule out any alternative aetiology (e.g., disease progression, other medications, or infections) |
| | **Grade 1**<br><br>(asymptomatic with laboratory [e.g., BNP] or cardiac imaging abnormalities) | No dose modifications required unless clinical suspicion is high, in which case hold study drug/study regimen dose during diagnostic work-up for other etiologies. If study drug/study regimen is held, resume after complete resolution to Grade 0. | **For Grade 1 (no definitive findings):**<br><br>-Monitor and closely follow up in 2 to 4 days for clinical symptoms, BNP, cardiac enzymes, ECG, ECHO, pulse oximetry (resting and exertion), and laboratory work-up as clinically indicated.<br><br>-Consider using steroids if clinical suspicion is high. |

| Adverse Events | Severity Grade of the Event (NCI CTCAE version 4.0) | Dose Modifications | Toxicity Management |
|---|---|---|---|
| | **Grade 2, 3 or 4**<br>(Grade 2: Symptoms with mild to moderate activity or exertion)<br>(Grade 3: Severe with symptoms at rest or with minimal activity or exertion; intervention indicated)<br>(Grade 4: Life-threatening consequences; urgent<br>intervention indicated (e.g., continuous IV therapy or mechanical hemodynamic support)) | -If Grade 2 - Hold study drug/study regimen dose until resolution to Grade 0. If toxicity rapidly improves to Grade 0, then the decision to reinitiate study drug/study regimen will be based upon treating physician's clinical judgment and after completion of steroid taper. If toxicity does not rapidly improve, permanently discontinue study drug/study regimen.<br>If Grade 3-4, permanently discontinue study drug/study regimen. | **For Grade 2-4:**<br>−Monitor symptoms daily, hospitalize.<br>−Promptly start IV methylprednisolone 2 to 4 mg/kg/day or equivalent after Cardiology consultation has determined whether and when to complete diagnostic procedures including a cardiac biopsy.<br>−Supportive care (e.g., oxygen).<br>−If no improvement within 3 to 5 days despite IV methylprednisolone at 2 to 4 mg/kg/day, promptly start immunosuppressive therapy such as TNF inhibitors (e.g., infliximab at 5 mg/kg every 2 weeks). Caution: It is important to rule out sepsis and refer to infliximab label for general guidance before using infliximab.<br>−Once the patient is improving, gradually taper steroids over ≥28 days and consider prophylactic antibiotics, antifungals, or anti-PJP treatment (refer to current NCCN guidelines for treatment of cancer-related infections).[a] |
| **Myositis/ Polymyositis ("Poly/myositis" )** | **Any Grade** | **General Guidance** | **For Any Grade**<br>−Monitor patients for signs and symptoms of poly/myositis. Typically, muscle weakness/pain occurs in proximal muscles including upper arms, thighs, shoulders, hips, neck and back, but rarely affects the extremities including hands and fingers; also difficulty breathing and/or trouble swallowing can occur and progress rapidly. Increased general feelings of tiredness and fatigue may occur, and there can be new-onset falling, difficulty getting up from a fall, and trouble climbing stairs, standing up from a |

| Adverse Events | Severity Grade of the Event (NCI CTCAE version 4.0) | Dose Modifications | Toxicity Management |
|---|---|---|---|
| | | | seated position, and/or reaching up.<br>−If poly/myositis is suspected, a Neurology consultation should be obtained early, with prompt guidance on diagnostic procedures. Myocarditis may co-occur with poly/myositis; refer to guidance under Myocarditis. Given breathing complications, refer to guidance under Pneumonitis/ILD.<br>Given possibility of an existent (but previously unknown) autoimmune disorder, consider Rheumatology consultation.<br>−Consider, as necessary, discussing with the study physician.<br>−Initial work-up should include clinical evaluation, creatine kinase, aldolase, LDH, BUN/creatinine, erythrocyte sedimentation rate or C-reactive protein level, urine myoglobin, and additional laboratory work-up as indicated, including a number of possible rheumatological/antibody tests (i.e., consider whether a rheumatologist consultation is indicated and could guide need for rheumatoid factor, antinuclear antibody, anti-smooth muscle, antisynthetase [such as anti-Jo-1], and/or signal-recognition particle antibodies). Confirmatory testing may include electromyography, nerve conduction studies, MRI of the muscles, and/or a muscle biopsy. Consider Barium swallow for evaluation of dysphagia or dysphonia.<br>Patients should be thoroughly evaluated to rule out any alternative aetiology (e.g., disease progression, other medications, or infections). |

| Adverse Events | Severity Grade of the Event (NCI CTCAE version 4.0) | Dose Modifications | Toxicity Management |
|---|---|---|---|
| | **Grade 1**<br>(mild pain) | No dose modifications | **For Grade 1:**<br>−Monitor and closely follow up in 2 to 4 days for clinical symptoms and initiate evaluation as clinically indicated.<br>−Consider Neurology consult.<br>−Consider, as necessary, discussing with the study physician. |
| | **Grade 2**<br>(moderate pain associated with weakness; pain limiting instrumental activities of daily living [ADLs]) | Hold study drug/study regimen dose until resolution to Grade ≤1.<br>-Permanently discontinue study drug/study regimen if it does not resolve to Grade ≤1 within 30 days or if there are signs of respiratory insufficiency. | **For Grade 2:**<br>−Monitor symptoms daily and consider hospitalization.<br>−Obtain Neurology consult, and initiate evaluation.<br>−Consider, as necessary, discussing with the study physician.<br>−If clinical course is rapidly progressive (particularly if difficulty breathing and/or trouble swallowing), promptly start IV methylprednisolone 2 to 4 mg/kg/day systemic steroids along with receiving input from Neurology consultant<br>−If clinical course is not rapidly progressive, start systemic steroids (e.g., prednisone 1 to 2 mg/kg/day PO or IV equivalent); if no improvement within 3 to 5 days, continue additional work up and start treatment with IV methylprednisolone 2 to 4 mg/kg/day<br>−If after start of IV methylprednisolone at 2 to 4 mg/kg/day there is no improvement within 3 to 5 days, consider start of immunosuppressive therapy such as TNF inhibitors (e.g., infliximab at 5 mg/kg every 2 weeks). Caution: It is important to rule out sepsis and refer to infliximab label for general guidance before using infliximab.<br>−Once the patient is improving, gradually taper steroids over ≥28 days |

| Adverse Events | Severity Grade of the Event (NCI CTCAE version 4.0) | Dose Modifications | Toxicity Management |
|---|---|---|---|
| | | | and consider prophylactic antibiotics, antifungals, or anti-PJP treatment (refer to current NCCN guidelines for treatment of cancer-related infections).[a] |
| | **Grade 3 or 4**<br>(pain associated with severe weakness; limiting self-care ADLs) | **For Grade 3:**<br>Hold study drug/study regimen dose until resolution to Grade ≤1.<br>Permanently discontinue study drug/study regimen if Grade 3 imAE does not resolve to Grade ≤1 within 30 days or if there are signs of respiratory insufficiency.<br>**For Grade 4:**<br>-Permanently discontinue study drug/study regimen. | **For Grade 3 or 4 (severe or life-threatening events):**<br>−Monitor symptoms closely; recommend hospitalization.<br>−Obtain Neurology consult, and complete full evaluation.<br>−Consider, as necessary, discussing with the study physician.<br>−Promptly start IV methylprednisolone 2 to 4 mg/kg/day systemic steroids along with receiving input from Neurology consultant.<br>−If after start of IV methylprednisolone at 2 to 4 mg/kg/day there is no improvement within 3 to 5 days, consider start of immunosuppressive therapy such as TNF inhibitors (e.g., infliximab at 5 mg/kg every 2 weeks). Caution: It is important to rule out sepsis and refer to infliximab label for general guidance before using infliximab.<br>−Consider whether patient may require IV IG, plasmapheresis.<br>−Once the patient is improving, gradually taper steroids over ≥28 days and consider prophylactic antibiotics, antifungals, or anti-PJP treatment (refer to current NCCN guidelines for treatment of cancer-related infections)[a] |

[a]ASCO Educational Book 2015 “Managing Immune Checkpoint Blocking Antibody Side Effects” by Michael Postow MD.

[b]FDA Liver Guidance Document 2009 Guidance for Industry: Drug Induced Liver Injury – Premarketing Clinical Evaluation.

AChE Acetylcholine esterase; ADL Activities of daily living; AE Adverse event; ALP Alkaline phosphatase test; ALT Alanine aminotransferase; AST Aspartate aminotransferase; BUN Blood urea nitrogen; CT Computed tomography; CTCAE Common Terminology Criteria for Adverse Events; ILD Interstitial lung disease; imAE immune-mediated adverse event; IG Immunoglobulin; IV Intravenous; GI Gastrointestinal; LFT Liver function tests; LLN Lower limit of normal; MRI  Magnetic resonance imaging; NCI  National Cancer Institute; NCCN  National Comprehensive Cancer Network; PJP Pneumocystis jirovecii pneumonia (formerly known as Pneumocystis carinii pneumonia); PO  By mouth; T3  Triiodothyronine; T4 Thyroxine; TB Total bilirubin; TNF Tumor necrosis factor; TSH Thyroid-stimulating hormone; ULN Upper limit of normal.

## Infusion-Related Reactions

| Severity Grade of the Event (NCI CTCAE version 4.03) | Dose Modifications | Toxicity Management |
|---|---|---|
| **Any Grade** | General Guidance | **For Any Grade:**<br>−Manage per institutional standard at the discretion of investigator.<br>−Monitor patients for signs and symptoms of infusion-related reactions (e.g., fever and/or shaking chills, flushing and/or itching, alterations in heart rate and blood pressure, dyspnea or chest discomfort, or skin rashes) and anaphylaxis (e.g., generalized urticaria, angioedema, wheezing, hypotension, or tachycardia). |
| **Grade 1 or 2** | **For Grade 1:**<br>The infusion rate of study drug/study regimen may be decreased by 50% or temporarily interrupted until resolution of the event.<br>**For Grade 2:**<br>The infusion rate of study drug/study regimen may be decreased 50% or temporarily interrupted until resolution of the event.<br>Subsequent infusions may be given at 50% of the initial infusion rate. | **For Grade 1 or 2:**<br>−Acetaminophen and/or antihistamines may be administered per institutional standard at the discretion of the investigator.<br>−Consider premedication per institutional standard prior to subsequent doses.<br>−Steroids should not be used for routine premedication of Grade ≤2 infusion reactions. |
| **Grade 3 or 4** | **For Grade 3 or 4:**<br>Permanently discontinue study drug/study regimen. | **For Grade 3 or 4:**<br>−Manage severe infusion-related reactions per institutional standards (e.g., IM epinephrine, followed by IV diphenhydramine and ranitidine, and IV glucocorticoid). |

CTCAE Common Terminology Criteria for Adverse Events; IM intramuscular; IV intravenous; NCI National Cancer Institute.

## Non-Immune-Mediated Reactions

| Severity Grade of the Event (NCI CTCAE version 4.03) | Dose Modifications | Toxicity Management |
|---|---|---|
| **Any Grade** | Note: Dose modifications are not required for AEs not deemed to be related to study treatment (i.e., events due to underlying disease) or for laboratory abnormalities not deemed to be clinically significant. | Treat accordingly, as per institutional standard. |
| **Grade 1** | No dose modifications. | Treat accordingly, as per institutional standard. |
| **Grade 2** | Hold study drug/study regimen until resolution to ≤Grade 1 or baseline. | Treat accordingly, as per institutional standard. |
| **Grade 3** | Hold study drug/study regimen until resolution to ≤Grade 1 or baseline.<br><br>For AEs that downgrade to ≤Grade 2 within 7 days or resolve to ≤Grade 1 or baseline within 14 days, resume study drug/study regimen administration. Otherwise, discontinue study drug/study regimen. | Treat accordingly, as per institutional standard. |
| **Grade 4** | Discontinue study drug/study regimen (Note: For Grade 4 labs, decision to discontinue should be based on accompanying clinical signs/symptoms, the Investigator's clinical judgment, and consultation with the Sponsor.). | Treat accordingly, as per institutional standard. |

Note: As applicable, for early phase studies, the following sentence may be added: "Any event greater than or equal to Grade 2, please discuss with Study Physician."

AE Adverse event; CTCAE Common Terminology Criteria for Adverse Events; NCI National Cancer Institute.

## Supplementary Appendix 5: Prohibited concomitant medications for use during cisplatin therapy (arm 1)

- Simultaneous use of myelosuppressives or radiation will boost the effects of cisplatin's myelosuppressive activity. The occurrence of nephrotoxicity caused by cisplatin may be intensified by concomitant treatment with antihypertensives containing furosemide, hydralazine, diazoxide, and propranolol.
- Concomitant administration of nephrotoxic (e.g., cephalosporins, aminoglycosides or Amphotericin B or contrast media) or ototoxic (e.g., aminoglycosides) medicinal products will potentiate the toxic effect of cisplatin on the kidneys. During or after treatment with cisplatin caution is advised with predominantly renally eliminated substances. The following substances are prohibited:
    - Cytostatic agents such as bleomycin and methotrexate because of potentially reduced renal elimination
    - Cisplatin given in combination with bleomycin and vinblastine can lead to a Raynaud's phenomenon
    - Ifosfamide may increase renal toxicity when used with cisplatin or in patients who have previously been given cisplatin
    - Chelating agents like penicillamine may diminish the effectiveness of cisplatin
    - Cisplatin given with cyclosporine causes excessive immunosuppression, increasing the risk of lymphoproliferation

  Reduction of blood lithium values has been noticed in a few cases after treatment with cisplatin combined with bleomycin and etoposide. It is therefore recommended to monitor the lithium values. Furthermore, it may be required to adjust the dosage of allopurinol, colchicine, probenecid, or sulfinpyrazone if used together with cisplatin, since cisplatin causes an increase in serum uric acid concentration.
- Concomitant administration of ototoxic (e.g., aminoglycosides, loop diuretics) medicinal products will potentiate the toxic effect of cisplatin on auditory function. Except for patients receiving doses of cisplatin exceeding 60mg/m$^2$, whose urine secretion is less than 1000ml per 24 hours, no forced diuresis with loop diuretics should be applied in view of possible damage to the kidney tract and ototoxicity. Ifosfamide may increase hearing loss in combination with cisplatin.

- Yellow fever vaccine is strictly contraindicated because of the risk of fatal systemic vaccinal disease. In view of the risk of generalised illness; it is advisable to use an inactive vaccine if available.
- In the event of simultaneous use of oral anticoagulants, it is advisable to regularly check the INR.
- Simultaneous use of antihistamines, buclizine, cyclizine, loxapine, meclozine, phenothiazines, thioxanthenes or trimethobenzamides may mask ototoxicity symptoms (such as dizziness and tinnitus).
- Serum concentrations of anticonvulsive medicines may remain at sub-therapeutic levels during treatment with cisplatin. Cisplatin may reduce the absorption of phenytoin resulting in reduced epilepsy control when phenytoin is given as concurrent treatment. During cisplatin therapy starting a new anticonvulsant treatment with phenytoin is strictly contraindicated.
- Treatment with cisplatin prior to an infusion with paclitaxel may reduce the clearance of paclitaxel by 33% and therefore can intensify neurotoxicity.

Please note that cisplatin reacts with metallic aluminium to form a black precipitate of platinum. All aluminium containing IV sets, needles, catheters and syringes should be avoided.

## Supplementary Appendix 6: Prohibited concomitant medications for use with durvalumab (arm 5)

| Prohibited medication/class of drug | Usage |
|---|---|
| Immunosuppressive medications including, but not limited to, systemic corticosteroids at doses exceeding 10 mg/day of prednisone or equivalent, methotrexate, azathioprine, and tumour necrosis factor-α blockers | Should not be given during the study. (Use of immunosuppressive medications for the management of IP-related AEs or in patients with contrast allergies is acceptable. In addition, use of inhaled, topical, and intranasal corticosteroids is permitted. Temporary use of corticosteroids (up to two weeks) for concurrent illnesses [e.g., food allergies or CT scan contrast hypersensitivity] is acceptable upon discussion with the Investigator) |
| Herbal and natural remedies which may have immune-modulating effects | Should not be given concomitantly unless agreed by the sponsor |
| EGFR TKIs | Should not be given concomitantly.<br>Should be used with caution in the 90 days post last dose of durvalumab.<br>Increased incidences of pneumonitis (with third generation EGFR TKIs) and increased incidence of transaminase increases (with 1st generation EGFR TKIs) has been reported when durvalumab has been given concomitantly. |

# Supplementary Appendix 7: Definitions of adverse events

## Adverse Event

Any untoward medical occurrence in a patient or clinical trial subject administered a medicinal product and which does not necessarily have a causal relationship with this treatment.

Comment:

An AE can therefore be any unfavourable and unintended sign (including abnormal laboratory findings), symptom or disease temporally associated with the use of an investigational medicinal product, whether or not related to the investigational medicinal product.

## Adverse Reaction

All untoward and unintended responses to an IMP related to any dose administered.

Comment:

An AE judged by either the reporting investigator or Sponsor as having causal relationship to the IMP qualifies as an AR. The expression reasonable causal relationship means to convey in general that there is evidence or argument to suggest a causal relationship.

## Serious Adverse Event

Any untoward medical occurrence or effect that at any dose:

- Results in death unrelated to the original cancer
- Is life-threatening*
- Requires hospitalisation** or prolongation of existing inpatients' hospitalisation
- Results in persistent or significant disability or incapacity
- Is a congenital anomaly/birth defect
- Or is otherwise considered medically significant by the investigator***

Comments:

The term severe is often used to describe the intensity (severity) of a specific event. This is not the same as serious, which is based on patients/event outcome or action criteria.

* Life threatening in the definition of an SAE refers to an event in which the patient was at risk of death at the time of the event; it does not refer to an event that hypothetically might have caused death if it were more severe.

**Hospitalisation is defined as an unplanned, formal inpatient admission, even if the hospitalisation is a

precautionary measure for continued observation. Thus hospitalisation for protocol treatment (e.g. line insertion), elective procedures (unless brought forward because of worsening symptoms) or for social reasons (e.g. respite care) are not regarded as an SAE.

*** Medical judgment should be exercised in deciding whether an AE is serious in other situations. Important AEs that are not immediately life threatening or do not result in death or hospitalisation but may jeopardise the subject or may require intervention to prevent one of the other outcomes listed in the definition above, should be considered serious.

## Serious Adverse Reaction

An Adverse Reaction which also meets the definition of a Serious Adverse Event.

## Suspected Unexpected Serious Adverse Reaction

A SAR that is unexpected i.e. the nature, or severity of the event is not consistent with the Reference Safety Information.

A SUSAR should meet the definition of an AR, unexpected adverse reason (UAR) and SAR.

## Unexpected Adverse Reaction

An AR, the nature or severity of which is not consistent with the Reference Safety Information.

When the outcome of an AR is not consistent with the Reference Safety Information the AR should be considered unexpected.
